# LPLAT11/MBOAT7-driven phosphatidylinositol remodeling ensures radial glial cell integrity in developing neocortex

**DOI:** 10.1016/j.isci.2025.114248

**Published:** 2025-11-27

**Authors:** Yuki Ishino, Yusuke Kishi, Taiga Iwama, Naohiro Kuwayama, Hiroyuki Arai, Yukiko Gotoh, Junken Aoki, Nozomu Kono

**Affiliations:** 1 Laboratory of Health Chemistry, Graduate School of Pharmaceutical Sciences, The University of Tokyo, Tokyo 113-0033, Japan; 2 Institute for Quantitative Biosciences, The University of Tokyo, Tokyo 113-0032, Japan; 3 Laboratory of Molecular Biology, Graduate School of Pharmaceutical Sciences, The University of Tokyo, Tokyo 113-0033, Japan; 4 Laboratory of MicroEnvironment and Metabolic Health Science, Center for Disease Biology and Integrative Medicine, Faculty of Medicine, The University of Tokyo, Tokyo 113-0033, Japan; 5 International Research Center for Neurointelligence (WPI-IRCN), The University of Tokyo, Tokyo 113-0033, Japan

**Keywords:** Neuroscience, Developmental neuroscience, Cellular neuroscience

## Abstract

Phosphatidylinositol (PI) is highly enriched in arachidonic acid, an essential polyunsaturated fatty acid for brain development. This enrichment is mediated by the phospholipid remodeling enzyme lysophospholipid acyltransferase 11 (LPLAT11), also known as membrane-bound *O*-acyltransferase 7 (MBOAT7), whose deficiency causes microcephaly in both humans and mice. Here, we show that *Mboat7* deficiency impairs indirect neurogenesis in the developing neocortex by compromising radial glial cell (RGC) integrity, resulting in fewer layer II–V neurons. *Mboat7*-deficient RGCs exhibited decreased proliferation, impaired differentiation into intermediate progenitor cells, and increased apoptosis. These defects were preceded by Golgi apparatus rounding, reduced apical E-cadherin expression, and dysregulated apical detachment of RGCs. Moreover, the *Mboat7*-deficient cortex displayed reduced PI(4,5)P_2_ levels, and pharmacological inhibition of PI(4,5)P_2_ synthesis recapitulated the Golgi rounding observed in *Mboat7*-deficient RGCs. These findings suggest that compromised RGC integrity due to reduced PI(4,5)P_2_ levels, resulting from decreased arachidonic acid-containing PI, underlies the microcephaly associated with *MBOAT7* deficiency.

## Highlights

• *Mboat7* deficiency impairs radial glial cell integrity and indirect neurogenesis
• Radial glial cells lacking *Mboat7* display Golgi apparatus rounding
• PI(4,5)P_2_ levels decline in the *Mboat7*-deficient cortex at the onset of neurogenesis
• Blocking PI(4,5)P_2_ synthesis recapitulates Golgi rounding in radial glial cells

## Graphical abstract

**Figure:**
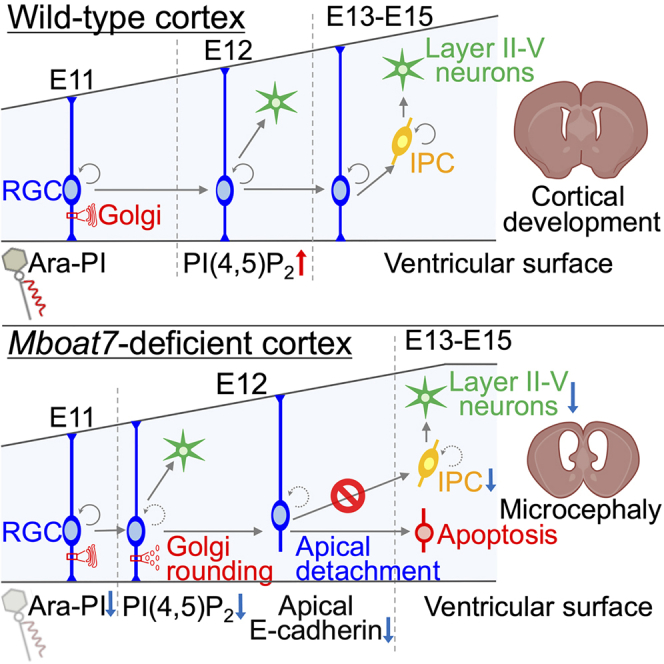

## Introduction

Phospholipids in biological membranes exhibit remarkable structural diversity due to variations in their polar head groups and fatty acyl chains, giving rise to over a thousand distinct phospholipid species in mammalian cells.^1^ This diversity is generated through the phospholipid remodeling pathway (also known as Lands’ cycle), in which newly synthesized phospholipids undergo deacylation by phospholipase A (PLA), followed by reacylation with specific fatty acids by lysophospholipid acyltransferases (LPLATs).^2^ Recent studies using LPLAT-deficient animal models have highlighted the significance of phospholipid remodeling in diverse physiological and pathological contexts, including development, lipoprotein synthesis, obesity, and hepatic steatosis.^3^^,^^4^^,^^5^^,^^6^^,^^7^^,^^8^^,^^9^^,^^10^

Among the phospholipid classes, the phosphatidylinositol (PI) class is unique in that it predominantly contains arachidonic acid at the *sn*-2 position, and phospholipid remodeling underlies the selective enrichment of PI with arachidonic acid.^11^^,^^12^ Lysophospholipid acyltransferase 11 (LPLAT11, also known as LPIAT1 or MBOAT7) is the remodeling enzyme that specifically incorporates arachidonic acid into PI.^13^ LPLAT11 exhibits its highest activity when arachidonoyl-CoA and lysoPI are used as the acyl donor and acceptor, respectively.^13^^,^^14^^,^^15^ Notably, patients harboring null mutations in *MBOAT7* show severe intellectual disability, epilepsy, autistic features, and microcephaly.^16^^,^^17^^,^^18^^,^^19^^,^^20^^,^^21^^,^^22^^,^^23^^,^^24^ Consistent with these findings, *Mboat7* knockout (KO) mice also exhibit microcephaly.^4^^,^^25^^,^^26^ Our previous study demonstrated that *Mboat7* KO mice display disorganized cortical lamination, delayed neuronal migration in the embryonic day 18.5 (E18.5) cortex, and an increased number of TUNEL-positive cells in the E14.5 cortex.^4^ Some studies also reported that *Mboat7* KO mice develop a deformed, domed-shaped head^25^^,^^26^ and hydrocephalus.^25^ More recently, Tang et al.,^27^ based on findings that lysoPI levels are elevated in the E13.5 brain and that mTOR activity is increased in delaminated neural progenitors in the E14.5 cortex of *Mboat7* KO mice, proposed that increased lysoPI activates mTOR signaling, leading to apoptosis and neuronal migration defects in *Mboat7* KO mice.

Microcephaly arises from a reduced number of neurons generated during embryonic development. In the developing murine cerebral cortex, neural stem cells (radial glial cells [RGCs]) generate neurons primarily through asymmetric divisions during early neurogenesis (E11–E13), a process called direct neurogenesis.^28^ During mid-corticogenesis (E13–E16), RGCs predominantly generate neurons via indirect neurogenesis, wherein RGCs first produce intermediate progenitor cells (IPCs) through asymmetric divisions, and IPCs subsequently differentiate into neurons after one or two rounds of division.^29^^,^^30^^,^^31^^,^^32^ Thus, RGCs play a pivotal role in corticogenesis, and their dysfunction leads to fewer neurons and microcephaly. While Tang et al.^27^ mainly analyzed abnormalities in IPCs of *Mboat7* KO mice, how *Mboat7* deficiency affects RGC functions remains to be elucidated.

In the present study, we investigated the cortical phenotype of global and neural-specific *Mboat7* KO mice during embryonic development, with particular focus on early neurogenic stages, and found that *Mboat7* deficiency compromised RGC integrity in the E12 cortex. *Mboat7*-deficient RGCs exhibited decreased proliferation, impaired differentiation into IPCs, and increased apoptosis, leading to a decrease in layer II–V neurons. Notably, prior to these defects, the Golgi apparatus became rounded, E-cadherin expression on the apical surface decreased, and apical detachment occurred in *Mboat7*-deficient RGCs. Furthermore, *Mboat7* deficiency led to a reduction in PI(4,5)P_2_ levels, and pharmacological inhibition of PI(4,5)P_2_ production induced Golgi rounding, along with decreased E-cadherin expression on the apical surface. Based on these findings, we propose that compromised RGC integrity due to decreased PI(4,5)P_2_ levels underlie the microcephaly observed in *Mboat7* deficiency.

## Results

### *Mboat7* KO mice exhibit reduced layer II–V neurons and increased cortical apoptosis

**Figure 1:**
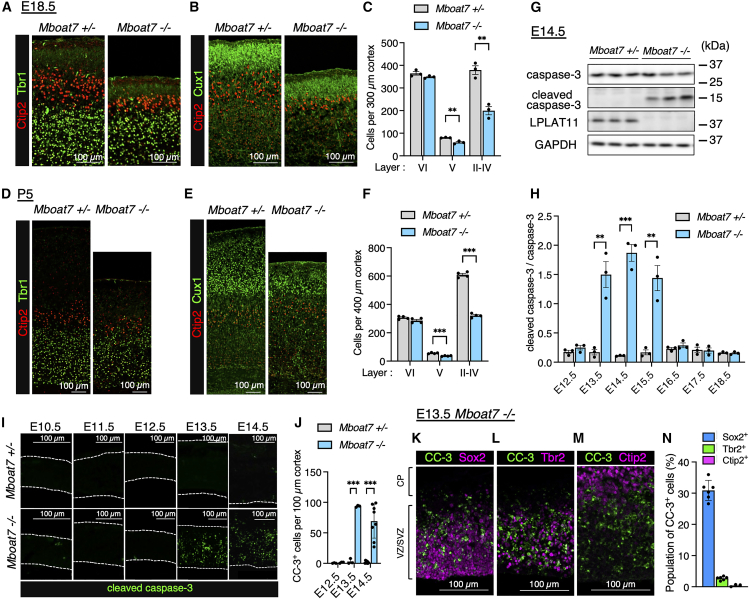
*Mboat7* KO mice exhibit reduced layer II–V neurons and increased cortical apoptosis during E13.5–E15.5 (A) Immunofluorescence staining of Tbr1 (green) and Ctip2 (red) in coronal sections of *Mboat7* heterozygous (+/−) and KO (−/−) mice at E18.5. (B) Immunofluorescence staining of Cux1 (green) and Ctip2 (red) in coronal sections of *Mboat7*^+/−^ and *Mboat7*^−/−^ mice at E18.5. (C) Quantitative analysis of layer VI (Tbr1^+^), V (Tbr1^−^ Ctip2^+^), and II–IV (Cux1^+^) neurons per area within 300-μm-wide bins (*n* = 3 embryos from two independent litters for each genotype). (D) Immunofluorescence staining of Tbr1 (green) and Ctip2 (red) in coronal sections of *Mboat7*^+/−^ and *Mboat7*^−/−^ mice at P5. (E) Immunofluorescence staining of Cux1 (green) and Ctip2 (red) in coronal sections of *Mboat7*^+/−^ and *Mboat7*^−/−^ mice at P5. (F) Quantitative analysis of layer VI (Tbr1^+^), V (Tbr1^−^ Ctip2^+^), and II–IV (Cux1^+^) neurons per area within 400-μm-wide bins (*n* = 4 embryos from two independent litters for each genotype). (G) Western blots of caspase-3 (the inactive precursor of apoptosis executioner), cleaved caspase-3 (an apoptosis marker), and LPLAT11 (a PI remodeling enzyme encoded by *Mboat7*) in the cortices of *Mboat7*^+/−^ and *Mboat7*^−/−^ mice at E14.5. GAPDH was used as a loading control. Molecular weight markers are shown to the right of the blots. (H) Evaluation of caspase-3 activation in the cortices of *Mboat7*^+/−^ and *Mboat7*^−/−^ mice from E12.5 to E18.5 (*n* = 3 embryos from two independent litters for each genotype). (I) Immunofluorescence staining for cleaved caspase-3 in the cortices of *Mboat7*^+/−^ and *Mboat7*^−/−^ mice from E10.5 to E14.5. Dotted lines show the apical and the basal surfaces of the cortex. (J) Quantitative analysis of cells positive for cleaved caspase-3 (CC-3) per area within 100-μm-wide bins (*n* = 3 embryos [E12.5 and E13.5] and *n* = 8 embryos [E14.5] from two independent litters for each genotype). (K–M) Double staining for cleaved caspase-3 (CC-3) and Sox2 (K), Tbr2 (L), and Ctip2 (M) in *Mboat7*^−/−^ mice cortex at E13.5. (N) Population analysis of cleaved caspase-3^+^ cells per area within 200-μm-wide bins of *Mboat7*^−/−^ mice cortex at E13.5 (*n* = 6 (K, L) and *n* = 3 (M) embryos from two independent litters). Sox2^+^ (blue), Tbr2^+^ (green), Ctip2^+^ (magenta). Data are shown as mean ± SEM; ∗∗*p* < 0.01, ∗∗∗*p* < 0.001; unpaired two-tailed Student’s *t* test and multiple *t* tests. CP, cortical plate; VZ, ventricular zone; SVZ, subventricular zone. Scale bars, 100 μm. See Data S1 for uncropped blots.

*Mboat7* KO mice have been reported to exhibit reduced cortical thickness with a reduced number of upper-layer neurons and temporarily restricted apoptosis around E14.5.^4^^,^^27^ Consistently, immunostaining of E18.5 cortices using markers for layer VI/subplate (Tbr1), layer VI/V (Ctip2), and layer II–IV neurons (Cux1) revealed a ∼50% reduction in the number of layer II–IV neurons in *Mboat7* KO mice compared to *Mboat7* heterozygous mice, while the number of layer VI neurons remained comparable (Figures 1A–1C). Additionally, the number of layer V neurons was modestly reduced to about 70%. Similar abnormalities were observed at postnatal day 5 (P5) when the migration of upper-layer neurons is complete, with some layer II–IV neurons displaying migration defects (Figures 1D–1F). During E13.5–E15.5, a critical period for layer II–V neuron generation, apoptosis was markedly increased in *Mboat7* KO mice, as indicated by immunoblot and immunostaining for cleaved caspase-3 (Figures 1G–1J). At this stage, the cortex mainly consists of RGCs, intermediate progenitor cells (IPCs), and neurons. To further characterize the apoptotic cells, *Mboat7* KO cortices were co-immunostained for cleaved caspase-3 along with markers for RGCs (Sox2), IPCs (Tbr2), and neurons (Ctip2). The results showed that apoptosis occurred primarily in RGCs and to a lesser extent in IPCs but not in neurons (Figures 1K–1N).

### Differentiation into IPCs and proliferation are impaired in *Mboat7* KO RGCs

**Figure 2:**
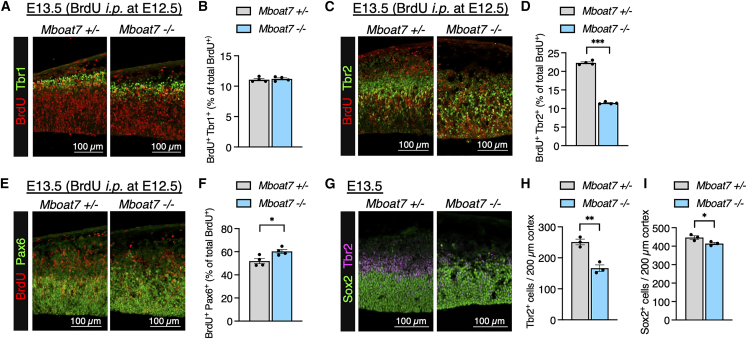
Differentiation of RGCs into IPCs is impaired in *Mboat7* KO mice (A) Double staining at E13.5 for BrdU (injected at E12.5) and Tbr1. (B) The rate of neuronal differentiation (percentage of BrdU^+^ Tbr1^+^ cells in BrdU^+^ cells) (*n* = 4 embryos from two independent litters for each genotype). (C) Double staining at E13.5 for BrdU (injected at E12.5) and Tbr2. (D) The rate of differentiation into IPCs (percentage of BrdU^+^ Tbr2^+^ cells in BrdU^+^ cells) (*n* = 4 embryos from two independent litters for each genotype). (E) Double staining at E13.5 for BrdU (injected at E12.5) and Pax6. (F) The rate of RGCs (percentage of BrdU^+^ Pax6^+^ cells in BrdU^+^ cells) (*n* = 4 embryos from two independent litters for each genotype). (G) Immunofluorescence staining of Sox2 (green) and Tbr2 (magenta) in coronal sections of *Mboat7*^+/−^ and *Mboat7*^−/−^ mice at E13.5. (H and I) Quantitative analysis of cells positive for Tbr2 (H) and Sox2 (I) per area within 200-μm-wide bins (*n* = 3 embryos from two independent litters for each genotype). Data are shown as mean ± SEM; ∗*p* < 0.05, ∗∗*p* < 0.01, ∗∗∗*p* < 0.001; unpaired two-tailed Student’s *t* test. Scale bars, 100 μm.

In addition to RGC apoptosis, the reduced number of neurons may result from impaired differentiation or proliferation of neural progenitors such as RGCs and IPCs. We first determined whether the differentiation of RGCs to neurons, i.e., direct neurogenesis, is affected in *Mboat7* KO mice. To this end, we administered bromodeoxyuridine (BrdU) intraperitoneally to E12.5 pregnant mice and analyzed the cortices of E13.5 embryos. Double staining for Tbr1 (a neuronal marker) and BrdU revealed that the proportion of Tbr1^+^ cells among BrdU^+^ cells was comparable between *Mboat7* heterozygous and KO mice (Figures 2A and 2B), indicating that direct neurogenesis remains unaffected in *Mboat7* KO mice. Next, to assess whether the differentiation of RGCs into IPCs (the initial step of indirect neurogenesis) is impaired in *Mboat7* KO mice, we performed immunostaining for Pax6 (an RGC marker), Tbr2, and BrdU in the cortices of BrdU-labeled E13.5 embryos. In *Mboat7* KO mice, the proportion of Tbr2^+^ cells among BrdU^+^ cells was reduced, whereas the proportion of Pax6^+^ cells among BrdU^+^ cells was increased (Figures 2C–2F), suggesting that differentiation of RGCs to IPCs was impaired. Quantification of cells positive for Tbr2 and Sox2 showed a significant decrease in IPCs and a slight decrease in RGCs in the *Mboat7* KO cortex (Figures 2G–2I). Given that apoptosis of IPCs was not prominent (Figures 1L and 1N), the marked decrease in IPCs was likely due to impaired differentiation of RGCs to IPCs.

**Figure 3:**
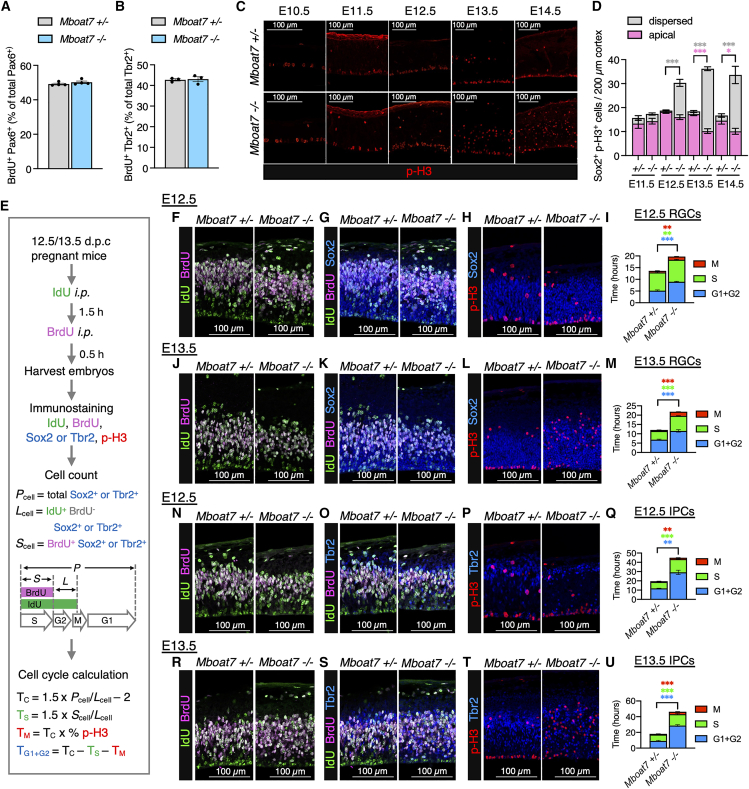
Proliferation of RGCs and IPCs is reduced in *Mboat7* KO mice (A) The rate of BrdU-labeled RGCs (percentage of BrdU^+^ Pax6^+^ cells in Pax6^+^ cells) (*n* = 4 embryos from two independent litters for each genotype). (B) The rate of BrdU-labeled IPCs (percentage of BrdU^+^ Tbr2^+^ cells in Tbr2^+^ cells) (*n* = 3 embryos from two independent litters for each genotype). (C) Immunofluorescence staining for phospho-histone H3 (p-H3) in the cortices of *Mboat7*^+/−^ and *Mboat7*^−/−^ mice from E10.5 to E14.5. (D) Quantitative analysis of apical and dispersed RGCs positive for p-H3 (Sox2^+^ p-H3^+^ cells) per area within 200-μm-wide bins (*n* = 3 embryos [E11.5, *Mboat7*^+/−^ and *Mboat7*^−/−^], *n* = 4 embryos [E12.5, *Mboat7*^+/−^], *n* = 5 embryos [E13.5, *Mboat7*^−/−^], *n* = 6 embryos [E12.5, *Mboat7*^−/−^], *n* = 7 embryos [E13.5 and E14.5, *Mboat7*^+/−^], and *n* = 8 embryos [E14.5, *Mboat7*^−/−^] from two independent litters). (E) IdU/BrdU injection paradigm to quantify total cell cycle, S-phase, M-phase, and G1+G2-phase lengths of RGCs and IPCs. A single IdU injection was performed at T = 0 h, followed by a single BrdU injection at T = 1.5 h, with harvest at T = 2 h. (F, J) Double staining of IdU (green) and BrdU (magenta) in coronal sections of *Mboat7*^+/−^ and *Mboat7*^−/−^ mice at E12.5 (F) and E13.5 (J). (G, K) Triple staining of IdU (green), BrdU (magenta), and Sox2 (blue) in coronal sections of *Mboat7*^+/−^ and *Mboat7*^−/−^ mice at E12.5 (G) and E13.5 (K). (H, L) Double staining of Sox2 (blue) and p-H3 (red) in coronal sections of *Mboat7*^+/−^ and *Mboat7*^−/−^ mice at E12.5 (H) and E13.5 (L). (I, M) Quantification of G1+G2-phase (blue), S-phase (green), M-phase (red), and total cell-cycle (full bar) lengths in *Mboat7*^+/−^ and *Mboat7*^−/−^ mice at E12.5 (*n* = 3 embryos (I) and *n* = 4 embryos (M) from two independent litters for each genotype). (N, R) Double staining of IdU (green) and BrdU (magenta) in coronal sections of *Mboat7*^+/−^ and *Mboat7*^−/−^ mice at E12.5 (N) and E13.5 (R). (O, S) Triple staining of IdU (green), BrdU (magenta), and Tbr2 (blue) in coronal sections of *Mboat7*^+/−^ and *Mboat7*^−/−^ mice at E12.5 (O) and E13.5 (S). (P, T) Double staining of Tbr2 (blue) and p-H3 (red) in coronal sections of *Mboat7*^+/−^ and *Mboat7*^−/−^ mice at E12.5 (P) and E13.5 (T). (Q, U) Quantification of G1+G2-phase (blue), S-phase (green), M-phase (red), and total cell-cycle (full bar) lengths in *Mboat7*^+/−^ and *Mboat7*^−/−^ mice at E12.5 (*n* = 3 embryos (Q), *n* = 5 embryos [(U), *Mboat7*^+/−^], and *n* = 4 embryos [(U), *Mboat7*^−/−^] from two independent litters). Data are shown as mean ± SEM; ∗*p* < 0.05, ∗∗*p* < 0.01, ∗∗∗*p* < 0.001; multiple *t* tests. The color of the asterisks corresponds to the color of the respective groups in the graph. Scale bars, 100 μm. See also Figure S1.

We next evaluated the proliferation of RGCs and IPCs. BrdU labeling experiments showed that the proportions of BrdU-labeled RGCs and IPCs were each comparable between *Mboat7* heterozygous and KO mice (Figures 3A and 3B). However, immunostaining for phospho-Histone H3 (p-H3), a marker for M-phase cells, showed a significant increase in the number of p-H3-positive RGCs in the E12.5-E14.5 cortices of *Mboat7* KO mice (Figures 3C and 3D), suggesting that the M phase is prolonged in *Mboat7* KO RGCs. To examine the cell-cycle length of RGCs and IPCs, we performed iododeoxyuridine (IdU) and BrdU double labeling,^33^ which can measure total cell-cycle length and S-phase duration (Figure 3E). The M-phase duration was also calculated using the total cell-cycle length and the proportion of p-H3^+^ cells in RGCs, while the combined duration of the G1 and G2 phases was taken as the total cell-cycle length minus the S and M phases. At E12.5 and E13.5, the overall cell cycle was prolonged in *Mboat7* KO RGCs (Figures 3F–3M), with a notable increase in M-phase proportion (Figures S1A and S1B). Similarly, the cell cycle of IPCs was prolonged (Figures 3N–3U), although the changes in the proportion of the IPCs in the M-phase proportion were less pronounced than they were in RGCs (Figures S1C and S1D). Thus, the proliferation of both RGCs and IPCs is impaired in *Mboat7* KO mice, and M-phase duration is prolonged in *Mboat7* KO RGCs.

**Figure 4:**
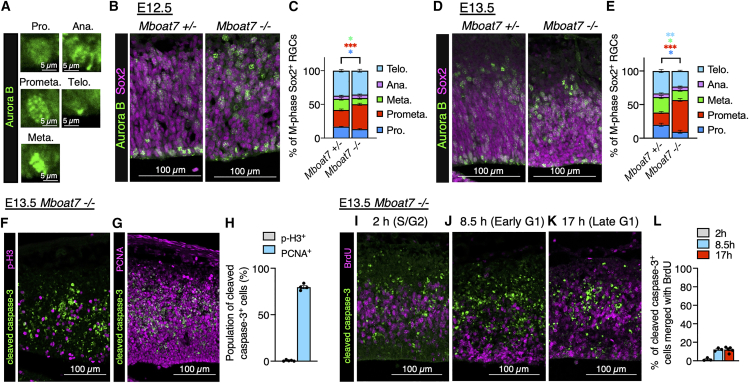
Prometaphase duration is prolonged in *Mboat7* KO RGCs (A) Immunofluorescence staining for Aurora B (green) shows phase-specific localization patterns of Aurora B in cells in prophase (Pro.), prometaphase (Prometa.), metaphase (Meta.), anaphase (Ana.), and telophase (Telo.) in the E13.5 cortex. (B) Immunofluorescence staining of Aurora B (green) and Sox2 (magenta) in coronal sections of *Mboat7*^+/−^ and *Mboat7*^−/−^ mice at E12.5. (C) Quantification of the percentage of Aurora B^+^ mitotic RGCs (Sox2^+^) across different mitotic phases in *Mboat7*^+/−^ and *Mboat7*^−/−^ mice at E12.5 (*n* = 5 embryos [*Mboat7*^+/−^] and *n* = 6 embryos [*Mboat7*^−/−^] from two independent litters). (D) Immunofluorescence staining of Aurora B (green) and Sox2 (magenta) in coronal sections of *Mboat7*^+/−^ and *Mboat7*^−/−^ mice at E13.5. (E) Quantification of the percentage of Aurora B^+^ mitotic RGCs (Sox2^+^) across different mitotic phases in *Mboat7*^+/−^ and *Mboat7*^−/−^ mice at E13.5 (*n* = 4 embryos [*Mboat7*^+/−^] and *n* = 6 embryos [*Mboat7*^−/−^] from two independent litters). (F, G) Double staining for cleaved caspase-3 (green) and either p-H3 (F, magenta) or PCNA (G, magenta) in the E13.5 cortices of *Mboat7*^−/−^ mice. (H) Quantitative analysis of cleaved caspase-3^+^ cells positive for p-H3 or PCNA in (F, G) (*n* = 5 embryos (F) and *n* = 4 embryos (G) from two independent litters). (I–K) Double staining for cleaved caspase-3 (green) and BrdU (magenta) in the E13.5 cortices of *Mboat7*^−/−^ mice, labeled for 2 h (I, the S/G2 phase), 8.5 h (J, the early G1 phase), and 17 h (K, the late G1 phase) in BrdU labeling. (L) Quantitative analysis of cleaved caspase-3^+^ cells positive for BrdU in (I–K) (*n* = 3 embryos (I, J) and *n* = 5 embryos (K) from two independent litters). Data are shown as mean ± SEM; ∗*p* < 0.05, ∗∗*p* < 0.01, ∗∗∗*p* < 0.001; multiple *t* tests. The color of the asterisks corresponds to the color of the respective groups in the graph. Scale bars, 5 μm in (A) and 100 μm (others).

Prolonged M phase, especially prometaphase, causes apoptosis several hours after cell division, possibly at the G1 phase, in RGCs.^34^ To examine which stage within the M phase is affected in *Mboat7* KO mice, we analyzed mitotic RGCs at E12.5 and E13.5 using Aurora B staining, which distinguishes prophase, prometaphase, metaphase, anaphase, and telophase in the M phase^35^ (Figure 4A). The proportion of prometaphase RGCs was increased in *Mboat7* KO mice (Figures 4B–4E). Moreover, cleaved caspase-3^+^ cells did not merge with p-H3 but merged with proliferating cell nuclear antigen (PCNA), a marker for proliferating cells (Figures 4F–4H), suggesting that proliferating cells outside of the M phase undergo apoptosis. To identify the cell-cycle phase of the apoptotic cells, BrdU was injected 2, 8.5, and 17 h before harvesting the cortex at E13.5 to label S/G2-, early G1-, and late G1-phase cells, respectively, followed by cleaved caspase-3 and BrdU staining.^36^ Under all conditions, cleaved caspase-3 rarely overlapped with BrdU (Figures 4I–4L), indicating that BrdU-positive RGCs do not undergo apoptosis. Since BrdU is incorporated during the S phase, this suggests that apoptosis occurs before cells enter the S phase. Combined with the finding that apoptosis does not occur during the M phase (Figures 4F and 4H), it is likely that apoptosis occurs in G1-phase cells that fail to enter the S phase.

### E-cadherin expression at the apical surface is perturbed in *Mboat7* KO RGCs

**Figure 5:**
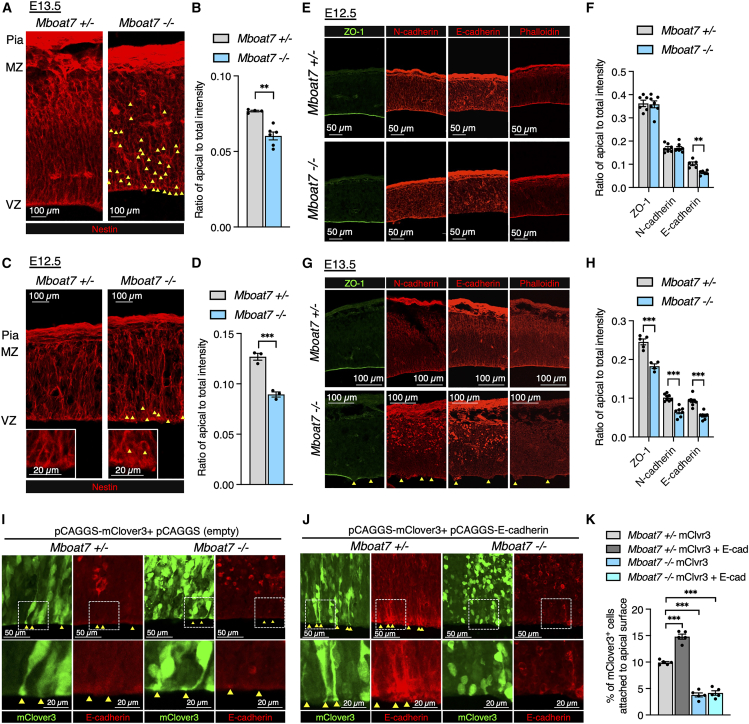
E-cadherin expression at the apical surface is perturbed in the cortex of *Mboat7* KO mice (A, C) Immunofluorescence staining for Nestin in the cortices of *Mboat7*^+/−^ and *Mboat7*^−/−^ mice at E13.5 (A) and E12.5 (C). Pia, pial surface; MZ, marginal zone; VZ, ventricular zone. Arrowheads show honeycomb-like pattern. (B, D) Ratio of apical intensity against total intensity from VZ to MZ is shown for Nestin at E13.5 (B) and E12.5 (D) (*n* = 3 embryos [(B), *Mboat7*^+/−^ and *Mboat7*^−/−^], *n* = 4 embryos [(D), *Mboat7*^+/−^], and *n* = 6 embryos [(D), *Mboat7*^−/−^] from two independent litters). (E, G) Immunofluorescence staining for ZO-1, N-cadherin, E-cadherin, and phalloidin in the cortices of *Mboat7*^+/−^ and *Mboat7*^−/−^ mice at E12.5 (E) and E13.5 (G). Arrowheads show the disruption of the ventricular wall. (F, H) Ratio of apical intensity against total intensity from VZ to MZ is shown for ZO-1, N-cadherin, and E-cadherin at E12.5 (F) and E13.5 (H) [(F) ZO-1, *n* = 6 (*Mboat7*^+/−^) and *n* = 7 embryos (*Mboat7*^−/−^); N-cadherin, *n* = 7 embryos (*Mboat7*^+/−^) and *n* = 8 embryos (*Mboat7*^−/−^); E-cadherin, *n* = 5 embryos (*Mboat7*^+/−^) and *n* = 6 embryos (*Mboat7*^−/−^) from two independent litters, (H) ZO-1, *n* = 5 (*Mboat7*^+/−^) and *n* = 4 embryos (*Mboat7*^−/−^); N-cadherin, *n* = 8 embryos (*Mboat7*^+/−^) and *n* = 7 embryos (*Mboat7*^−/−^); E-cadherin, *n* = 8 embryos (*Mboat7*^+/−^) and *n* = 7 embryos (*Mboat7*^−/−^) from two independent litters]. (I, J) pCAGGS-mClover3 and either pCAGGS empty vector (I) or pCAGGS-E-cadherin vector (J) were introduced into RGCs by *in utero* electroporation at E12.5. The E13.5 cortices were immunostained for E-cadherin (red). Arrowheads show mClover3^+^ RGCs with apical processes extending from the soma to the ventricular wall. Lower panels show zoomed-in views of the area within the dotted frame. (K) Percentage of mClover3^+^ cells attached to apical surface in the ventricular zone within 200-μm-wide bins (*n* = 5 embryos from two independent litters for each group). Data are shown as mean ± SEM; ∗∗*p* < 0.01, ∗∗∗*p* < 0.001; unpaired two-tailed Student’s *t* test (B), unpaired two-tailed Welch’s *t* test (D), multiple *t* tests (F, H), and one-way ANOVA with Tukey’s post hoc test (K). Scale bars, 100 μm in (A, C, G); 50 μm in (E, I, J); 20 μm in enlarged figures in (C, I, J).

RGCs constantly extend their processes radially, maintaining adhesion to both the apical and basal surfaces of the cortex. Nuclei of RGCs migrate basally in the G1/S phase and apically in the G2/M phase.^37^ Consequently, RGCs divide at the apical surface. In *Mboat7* KO mice, M-phase RGCs were dispersed throughout the ventricular/subventricular zone (Figures 3C and 3D), resembling the phenotype observed when apical processes lose their attachment to the ventricular surface.^38^^,^^39^ To examine the apical process of RGCs in *Mboat7* KO mice, we immunostained E12.5 and E13.5 cortices for Nestin, which is strongly expressed in the processes of RGCs. In E13.5 *Mboat7* heterozygous mice, RGC processes extended radially from the apical surface to the basal surface, with strong Nestin staining at the apical surface (Figure 5A). In contrast, *Mboat7* KO mice exhibited weak Nestin staining at the apical surface (Figures 5A and 5B), and their radial fiber arrangement was disordered (Figure 5A, arrowheads). Similar results, albeit to a lesser extent, were obtained in the E12.5 cortices of *Mboat7* KO mice (Figures 5C and 5D). These findings suggest that *Mboat7* KO RGCs lost contact with and detached from the apical surface.

RGCs adhere to the apical surface through adherens junctions composed of N-cadherin, E-cadherin, and ZO-1. In E12.5 *Mboat7* KO mice, E-cadherin expression at the apical surface was reduced, whereas the expressions of ZO-1 and N-cadherin, as well as actin filament organization at the ventricular wall, remained comparable to that in littermate controls (Figures 5E and 5F). However, in E13.5 *Mboat7* KO mice, the apical expressions of not only E-cadherin but also N-cadherin and ZO-1 were reduced (Figures 5G and 5H), along with disruption of the ventricular zone (Figure 5G, arrowheads).

To determine whether the reduced E-cadherin expression at the apical surface in *Mboat7* KO mice results from a decrease in total expression or a defect in intracellular localization, we introduced mClover3 and E-cadherin expression vectors into RGCs by *in utero* electroporation at E12.5 and analyzed the E13.5 cortex. In *Mboat7* heterozygous mice, co-transfection with mClover3 and E-cadherin expression vectors increased the levels of E-cadherin at the apical surface and the number of mClover3-positive RGC processes attached to the apical surface (Figures 5I–5K). In *Mboat7* KO mice, however, E-cadherin was not increased at the apical surface but instead accumulated in the soma (Figure 5J), and the number of apical processes attached to the ventricular surface was not increased (Figure 5K). Thus, in *Mboat7* KO RGCs, E-cadherin overexpression failed to restore its apical expression, suggesting that localization/trafficking of E-cadherin to the apical surface is impaired.

### *Mboat7* KO RGCs exhibit abnormal Golgi morphology

**Figure 6:**
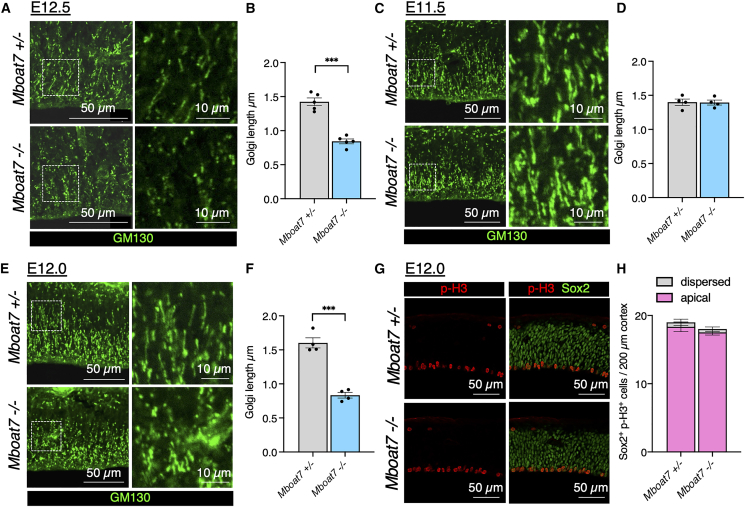
*Mboat7* deficiency causes abnormal morphology of the Golgi apparatus in the cortex (A) Immunofluorescence staining for GM130 in the cortices of *Mboat7*^+/−^ and *Mboat7*^−/−^ mice at E12.5. The right panels show zoomed-in views of the area within the dotted frame. (B) Measurement of the lengths of the average GM130^+^ Golgi apparatus in the ventricular zone within 50-μm-wide bins (*n* = 5 embryos from two independent litters for each genotype). (C) Immunofluorescence staining for GM130 in the cortices of *Mboat7*^+/−^ and *Mboat7*^−/−^ mice at E11.5. The right panels show zoomed-in views of the area within the dotted frame. (D) Measurement of the lengths of the average GM130^+^ Golgi apparatus in the ventricular zone within 50-μm-wide bins (*n* = 4 embryos from two independent litters for each genotype). (E) Immunofluorescence staining for GM130 in the cortices of *Mboat7*^+/−^ and *Mboat7*^−/−^ mice at E12.0. The right panels show zoomed-in views of the area within the dotted frame. (F) Measurement of the average lengths of the GM130^+^ Golgi apparatus in the ventricular zone within 50-μm-wide bins (*n* = 4 embryos from two independent litters for each genotype). (G) Double staining for p-H3 (red) and Sox2 (green) in the cortices of *Mboat7*^+/−^ and *Mboat7*^−/−^ mice at E12.0. (H) Quantitative analysis of apical and dispersed RGCs positive for p-H3 (Sox2^+^ and p-H3^+^ cells) per area within 200-μm-wide bins (*n* = 6 embryos from two independent litters for each genotype). Data are shown as mean ± SEM; ∗∗∗*p* < 0.001; unpaired two-tailed Welch’s *t* test. Scale bars, 10 μm [enlarged figures in (A, C, E)] and 50 μm (others). See also Figure S2.

The Golgi apparatus plays a pivotal role in E-cadherin trafficking.^40^^,^^41^^,^^42^ In RGCs, the Golgi apparatus is located exclusively in the apical process and extends along the apico-basal axis as the nucleus ascends during cell-cycle progression.^43^ Immunostaining of the E12.5 cortex for GM130, a marker for the Golgi apparatus, showed that the Golgi apparatus exhibited an elongated morphology in *Mboat7* heterozygous mice, whereas it appeared rounded in *Mboat7* KO mice (Figure 6A). Quantification of Golgi length in the ventricular zone confirmed a significant reduction in *Mboat7* KO mice (Figure 6B). At E11.5, however, the Golgi apparatus in *Mboat7* KO cortices did not yet exhibit a rounded morphology (Figures 6C and 6D). Further analysis of E12.0 cortices revealed that the Golgi was already rounded at this stage (Figures 6E and 6F). Notably, at this stage, p-H3^+^ RGCs remained attached to the apical surface in *Mboat7* KO mice cortices (Figures 6G and 6H), indicating that Golgi rounding precedes apical detachment.

To examine whether *Mboat7* KO mice exhibit any changes before E11.5, we evaluated global gene expression in the cortices of *Mboat7* heterozygous and KO mice at E11.5 using RNA sequencing. No significant differences in gene expression were observed (Figure S2A), suggesting that *Mboat7* KO mice develop normally until E11.5. At E12.5, when morphological abnormalities become apparent, gene expression changes began to emerge in the cortices, with three uncharacterized genes exhibiting differential expression and a broader set of genes displaying non-significant but discernible changes in expression levels (Figure S2B).

### PI(4,5)P_2_ is decreased in the *Mboat7* KO cortex

**Figure 7:**
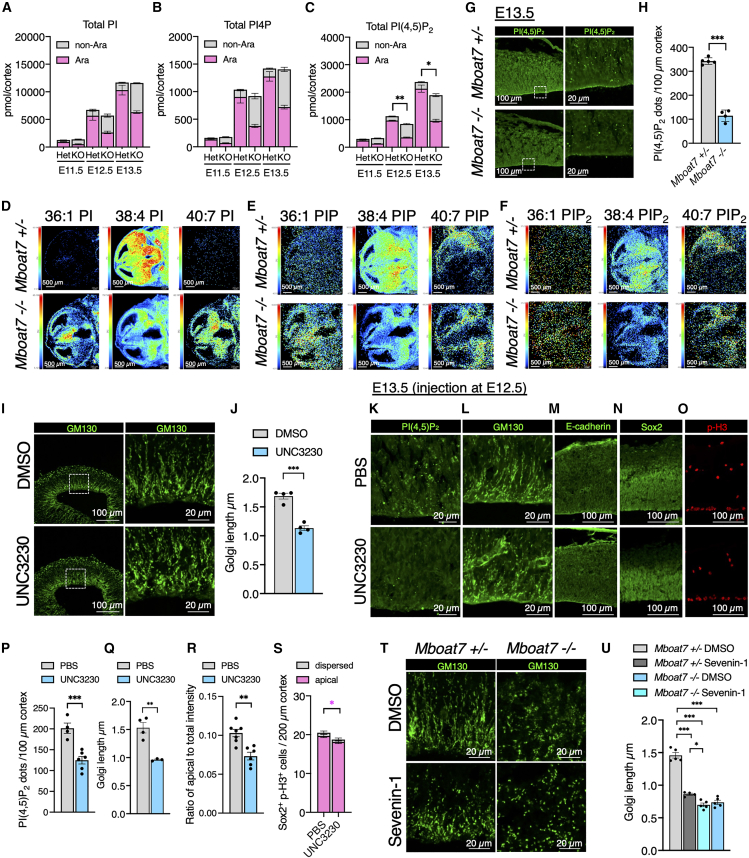
*Mboat7* KO cortex exhibits reduced PI(4,5)P_2_ levels, and pharmacological inhibition of PI(4,5)P_2_ synthesis induces Golgi rounding (A) Measurement of total PI in the cortices in *Mboat7*^+/−^ and *Mboat7*^−/−^ mice at E11.5–E13.5 using LC-MS/MS-based method (*n* = 3 embryos [E13.5, *Mboat7*^−/−^], *n* = 4 embryos [E11.5 and E12.5 *Mboat7*^+/−^], and *n* = 5 embryos [E12.5 *Mboat7*^−/−^ and E13.5 *Mboat7*^+/−^] from two independent litters). Peak areas are normalized by the area of the internal standard (25:0 PI). Ara and non-Ara indicate arachidonic acid-containing and non-arachidonic-acid-containing species, respectively. (B, C) Measurement of total PI4P (B) and PI(4,5)P_2_ (C) in the cortices in *Mboat7*^+/−^ and *Mboat7*^−/−^ mice at E11.5–E13.5 using SFC-MS/MS-based method (*n* = 3 embryos [E11.5, E12.5], *n* = 6 embryos [E13.5 *Mboat7*^−/−^], and *n* = 7 embryos [E13.5 *Mboat7*^+/−^] from two independent litters). Peak areas are normalized by the area of internal standard (37:4 PI4P or 37:4 PI(4,5)P_2_). (D–F) Imaging MS analysis of PI (D), PIP (E), and PIP_2_ (F) at E13.5 cortices of *Mboat7*^+/−^ and *Mboat7*^−/−^ mice. Signals were normalized by total ion current. (G) Immunofluorescence staining for PI(4,5)P_2_ in the cortices of *Mboat7*^+/−^ and *Mboat7*^−/−^ mice at E13.5. The right panels show zoomed-in views of the area within the dotted frame. (H) Quantitative analysis of PI(4,5)P_2_ positive dots per area within 100-μm-wide bins (*n* = 5 embryos [*Mboat7*^+/−^] and *n* = 4 embryos [*Mboat7*^−/−^] from two independent litters). (I) Immunofluorescence staining for GM130 in cultured E12.5 cortical hemispheres treated with DMSO or 1 μM PIPKIγ inhibitor (UNC3230). The Golgi apparatus is rounded in the hemispheres treated with UNC3230. The right panels show zoomed-in views of the area within the dotted frame. (J) Measurement of the lengths of the GM130^+^ Golgi apparatus in the ventricular zone within 50-μm-wide bins (*n* = 4 hemispheres [DMSO] and *n* = 4 hemispheres [UNC3230] from two independent litters and two independent experiments) in (I). (K–O) UNC3230 was administered into the ventricle of wild-type mice at E12.5. PBS (containing 0.1% DMSO) was administered as the control group. The E13.5 cortices were immunostained for PI(4,5)P_2_ (K), GM130 (L), E-cadherin (M), Sox2 (N), and p-H3 (O). (P) Quantitative analysis of PI(4,5)P_2_-positive dots per area within 100-μm-wide bins (*n* = 4 embryos [PBS] and *n* = 6 embryos [UNC3230] from two independent litters). (Q) Graph shows the lengths of the GM130^+^ Golgi apparatus in the ventricular zone within 50-μm-wide bins (*n* = 703 cells from 3 embryos [PBS] and *n* = 670 cells from 3 embryos [UNC3230] from two independent litters). (R) Ratio of apical intensity against total intensity from VZ to MZ is shown for E-cadherin (*n* = 7 embryos [PBS] and *n* = 6 embryos [UNC3230] from two independent litters). (S) Quantitative analysis of apical and dispersed RGCs positive for p-H3 (Sox2^+^ p-H3^+^ cells) per area within 200-μm-wide bins (*n* = 6 embryos [PBS] and *n* = 8 embryos [UNC3230] from two independent litters). (T) Immunofluorescence staining for GM130 in cultured E12.5 cortical hemispheres from *Mboat7*^+/−^ and *Mboat7*^−/−^ mice. Rounding of the Golgi apparatus was observed in *Mboat7*^+/−^ hemispheres treated with 10 μM LPLAT11 inhibitor (Sevenin-1). (U) Measurement of the lengths of the GM130^+^ Golgi apparatus in the ventricular zone within 50-μm-wide bins (*n* = 5 hemispheres [DMSO, *Mboat7*^+/−^], *n* = 4 hemispheres [Sevenin-1, *Mboat7*^+/−^], *n* = 5 hemispheres [DMSO, *Mboat7*^−/−^], and *n* = 4 hemispheres [Sevenin-1, *Mboat7*^−/−^] from two independent litters and two independent experiments). Data are shown as mean ± SEM; ∗*p* < 0.05, ∗∗*p* < 0.01, ∗∗∗*p* < 0.001; unpaired two-tailed Student’s *t* test (C, H, P, R, S), unpaired two-tailed Welch’s *t* test (J, Q), and one-way ANOVA with Tukey’s post hoc test (U). The color of the asterisks corresponds to the color of the respective groups in the graph. Scale bars, 20 μm [K, L, T, and enlarged figures in (G, I)]; 500 μm (D–F); 100 μm (others). See also Figures S3–S7 and Tables S1–S3.

Since gene expression changes in the E12.5 cortex of *Mboat7* KO mice were minimal, we reasoned that lipid alterations might contribute to the observed morphological abnormalities. In particular, we focused on phosphoinositides (PIPs), because defects in their metabolism have been reported to affect Golgi apparatus morphology.^44^ We employed liquid chromatography-mass spectrometry (LC-MS) to measure PI and supercritical fluid chromatography-mass spectrometry (SFC-MS)^45^ to measure PIPs in E11.5-E13.5 cortices. In *Mboat7* KO mice, PI with arachidonic acid (38:4 PI), the predominant species in *Mboat7* heterozygous mice, was significantly decreased (Figures S3A–S3C). On the other hand, PI with monounsaturated fatty acid (34:1, 34:2, 36:1, and 36:2 PI) and PI with docosapentaenoic acid (DPA) or docosahexaenoic acid (DHA) (38:6, 40:5, 40:6, and 40:7 PI) increased, in agreement with previous reports.^4^^,^^26^ PI4P and PI(4,5)P_2_, two major cellular PIPs, also exhibited changes in the molecular species similar to those observed in PI (Figures S3D–S3I). Notably, the total amount of PI(4,5)P_2_ started to decrease at E12.5 in *Mboat7* KO mice, while the total amounts of PI and PI4P remained unchanged (Figures 7A–7C). The reduction in PI(4,5)P_2_ was due to an insufficient increase in non-arachidonic-acid-containing PI(4,5)P_2_ species to compensate for the decrease in arachidonic-acid-containing PI(4,5)P_2_ species (Figures 7C, S3H, and S3I). Imaging MS analyses of E13.5 cortices yielded similar results but also revealed that molecular species of PI and PIPs were altered throughout the entire cortex of *Mboat7* KO mice (Figures 7D–7F). The composition of molecular species of phosphatidylserine (PS), phosphatidylcholine (PC), and phosphatidylethanolamine (PE) in the cortices of E11.5-E13.5 *Mboat7* KO mice showed little or no change compared with littermate controls (Figures S4A–S4I). PI(4,5)P_2_ staining of E13.5 cortices also showed a decrease in PI(4,5)P_2_ levels in the *Mboat7* KO cortex (Figures 7G and 7H).

To further examine whether PI(4,5)P_2_ is required for proper Golgi morphology in RGCs, we analyzed the effect of UNC3230, an inhibitor of PI4P 5-kinase type Iγ (PIPKIγ). We first applied UNC3230 to cortical hemisphere cultures, which closely mimic *in vivo* conditions and are useful for evaluating pharmacological treatments.^46^ Treatment of hemisphere cultures from wild-type mice with UNC3230 significantly reduced the total amount of PI(4,5)P_2_, primarily affecting PI(4,5)P_2_ species containing arachidonic acid and its analogous fatty acid, Mead acid (20:3), while exerting minimal effects on other species (Figures S5A and S5B). UNC3230 treatment induced Golgi apparatus rounding (Figures 7I and 7J), similar to the phenotype observed in *Mboat7* KO cortex and hemisphere cultures (Figures 6A and 6B, 7T, and 7U). Next, we administered UNC3230 into the lateral ventricle at E12.5 and immunostained E13.5 cortices with antibodies against PI(4,5)P_2_, GM130, E-cadherin, Sox2, and p-H3. This resulted in decreased PI(4,5)P_2_ levels, Golgi apparatus rounding, and reduced apical E-cadherin expression in the ventricular zone (Figures 7K–7M and 7P–7R). Detachment of RGCs from the ventricular wall was not observed, possibly due to the short duration following treatment (Figures 7N, 7O, and 7S). These findings suggest that reduced PI(4,5)P_2_ levels cause abnormal Golgi morphology and decreased E-cadherin expression at the apical surface.

To determine whether the enzymatic activity of LPLAT11, encoded by *Mboat7*, is required for proper Golgi morphology, we treated E12.5 *Mboat7* heterozygous hemispheres with Sevenin-1, an LPLAT11 inhibitor.^14^ SFC-MS analysis confirmed that Sevenin-1 treatment reduced the amount of PI with arachidonic acid (Figures S5C and S5E) and the total amount of PI(4,5)P_2_ (Figures S5D and S5F). Sevenin-1 treatment induced Golgi apparatus rounding, whereas it had no effect on the morphology of the Golgi apparatus in the *Mboat7* KO hemispheres (Figures 7T and 7U), underscoring the importance of LPLAT11 enzymatic activity.

Together, these results suggest that impaired remodeling of PI to arachidonic-acid-containing species due to *Mboat7* deficiency leads to a reduction in PI(4,5)P_2_ levels and consequently causes aberrant Golgi morphology and reduced E-cadherin expression at the apical surface in RGCs.

### Neural-specific *Mboat7* KO mice phenocopy global KO mice

To exclude the possibility that changes in systemic lipid metabolism in global *Mboat7* KO mice affect RGCs in a non-cell-autonomous manner, we generated neural-specific *Mboat7* KO mice (hereafter, *Mboat7*-Sox1-KO) by crossing *Mboat7*^*fl/fl*^ mice with Sox1-Cre transgenic mice, which drives the expression of Cre recombinase before E8.5 in the neuroepithelium.^47^^,^^48^ *Mboat7*-Sox1-KO mice exhibited microcephaly similar to that in *Mboat7* KO mice (Figures S6A and S6B). At E13.5, their cortices showed apoptosis and detachment of RGCs from the ventricular wall to the same extent as those observed in *Mboat7* KO cortices (Figures S6C–S6F), although RGC detachment was not observed at E12.5 (Figures S6E and S6F). The RGC detachment in *Mboat7*-Sox1-KO mice was not rescued by E-cadherin overexpression (Figures S6G–S6I). At E12.5, when RGC detachment was not yet apparent, *Mboat7*-Sox1-KO cortices exhibited rounding of the Golgi apparatus (Figures S6J and S6K), a phenotype similar to that observed in the E12.0 cortices of *Mboat7* KO mice (Figures 6E–6H). A significant alteration in the fatty acyl composition of PI and PIPs was observed in the cortices of *Mboat7*-Sox1-KO mice (Figures S7A–S7F), and the total amount of PI(4,5)P_2_ began to decrease at E12.5 (Figures S7G–S7I), as seen in the *Mboat7* KO cortices (Figure 7C). Taken together, these results indicate that *Mboat7*-Sox1-KO mice recapitulate almost all brain phenotypes observed in *Mboat7* KO mice. Notably, *Mboat7*-Sox1-KO mice died within a month after birth (Table S1), indicating that *Mboat7* deficiency in neural cells is responsible for the postnatal lethality of *Mboat7* KO mice.^4^

## Discussion

Polyunsaturated fatty acids, such as arachidonic acid and DHA, play crucial roles in embryonic brain development.^49^^,^^50^ Most PUFAs are present in the form bound to phospholipids in the brain. The fact that null mutations in *MBOAT7*, whose product LPLAT11 is responsible for incorporating arachidonic acid into PI, cause microcephaly both in humans and mice points to a crucial role for arachidonic acid in PI during cortical development.^4^^,^^27^ This is in sharp contrast to the fact that mice deficient in LPLAT12/LPCAT3 encoded by *Mboat5*, which is a major enzyme responsible for introducing arachidonic acid into PC, PE, and PS, show no significant brain phenotype.^5^^,^^51^ In the present study, we further explored the molecular mechanisms underlying microcephaly in *Mboat7* KO mice and found that *Mboat7* deficiency leads to several RGC abnormalities, including a decreased PI(4,5)P_2_ level, rounded Golgi morphology, apical detachment, reduced proliferation, impaired differentiation into IPCs, and increased apoptosis. A recent study proposed that impaired migration and increased apoptosis induced by lysoPI-mTOR signaling in IPCs underlie microcephaly associated with *MBOAT7* deficiency.^27^ Our findings complement and extend previous studies by uncovering the cellular and molecular basis of RGC abnormalities in *Mboat7* deficiency, providing a more profound understanding of the significance of arachidonic acid incorporation into PI during cortical development.

RGCs play a central role in neurogenesis during cortical development by directly differentiating into neurons (direct neurogenesis) or by generating IPCs, which subsequently produce neurons after one or two rounds of division (indirect neurogenesis).^28^ In the *Mboat7* KO cortex, the differentiation of RGCs into IPCs was markedly impaired, whereas direct neurogenesis appeared largely unaffected. This is consistent with the observation that the thickness of layers II–IV, which largely depends on indirect neurogenesis, was markedly reduced, while layer VI, which is primarily formed by direct neurogenesis, remained intact in the *Mboat7* KO cortex. Despite the impaired differentiation of RGCs to IPCs in the *Mboat7* KO cortex, the number of RGCs decreased rather than increased. This reduction can be attributed to both reduced proliferation and increased apoptosis of RGCs. In our analysis of RGC proliferation, we found that the M phase was prolonged, with a particular extension of the prometaphase in *Mboat7* KO RGCs. Notably, prolonged prometaphase in RGCs has previously been shown to suppress IPC generation and lead to apoptosis.^34^ Together, these findings suggest that prolonged prometaphase contributes to impaired IPC production and apoptosis in *Mboat7* KO RGCs.

RGCs are highly polarized along their apico-basal axis, and the architecture and integrity of their apical endfeet are maintained by cadherin-based adherens junctions. We found that the expressions of E-cadherin and N-cadherin at the ventricular surface of the *Mboat7* KO cortex began to decrease at E12.5 and E13.5, respectively. Concomitantly, M-phase RGCs dispersed throughout the ventricular and subventricular zones, suggesting that *Mboat7* KO RGCs aberrantly detach from the ventricular surface. The apical endfeet of RGCs serve as a niche for activating proliferation signaling, such as Wnt-β-catenin^52^^,^^53^ and Notch signaling.^54^^,^^55^ Upon differentiation of RGCs into neurons and IPCs, adherens junctions are disassembled in a controlled manner.^56^^,^^57^^,^^58^ However, their untimely loss has been shown to induce apoptosis.^38^^,^^59^ Thus, detachment of RGCs from the ventricular surface in the *Mboat7* KO cortex may also contribute to reduced proliferation and increased apoptosis.

Nestin staining revealed that RGC processes were disorganized in the *Mboat7* KO cortex, possibly due to RGC detachment. Since radially elongated RGC processes serve as scaffolds for neuronal migration,^32^ their disorganization may lead to neuronal migration defects. Indeed, impaired migration of IPCs and neurons has been observed in the cortex of *Mboat7* KO mice.^4^^,^^27^ Consistently, patients harboring null mutations in *MBOAT7* exhibit polymicrogyria,^27^ a condition thought to result from aberrant neuronal migration. Thus, impaired RGC integrity might contribute not only to microcephaly but also to the neuronal migration defects observed in *MBOAT7* deficiency.

*In utero* electroporation of the E-cadherin expression vector failed to restore the expression of E-cadherin at the apical surface in the *Mboat7* KO cortex. Instead, expressed E-cadherin was localized to the soma, suggesting that intracellular transport of E-cadherin to the apical membrane is disturbed in *Mboat7* KO RGCs. The Golgi apparatus of RGCs, which resides only in the apical process, contributes to the transport of apical membrane proteins.^60^ The Golgi apparatus of RGCs was rounded in the E12.0 cortex of *Mboat7* KO mice, where other cellular abnormalities were not noted. Thus, the rounding of the Golgi apparatus is an early cellular defect caused by *Mboat7* deficiency and may contribute to reduced levels of E-cadherin at the apical surface in *Mboat7* KO RGCs. The structure of the Golgi apparatus undergoes dynamic changes in coordination with the cell cycle.^61^^,^^62^ During interphase, it forms a ribbon-like structure, which is progressively disassembled into isolated ministacks before mitotic entry and subsequently into vesicles during prometaphase. These structural changes are essential for proper cell-cycle progression,^61^^,^^62^ as Golgi fragmentation and vesiculation are required for mitotic entry^63^^,^^64^ and bipolar spindle formation,^65^ respectively. Although both abnormal Golgi morphology and cell-cycle defects were observed in *Mboat7* KO RGCs, their causal relationship remains unclear and warrants further investigation.

Along with the abnormal Golgi morphology, we found that the level of PI(4,5)P_2_ was decreased in the E12.5 cortex of *Mboat7* KO mice. PI(4,5)P_2_ is critical for the trafficking of membrane proteins, including E-cadherin.^66^^,^^67^^,^^68^^,^^69^ For example, the interaction of Exo70 with PI(4,5)P_2_ is required for targeting of the exocyst complex to the plasma membrane, where it facilitates the tethering of post-Golgi secretory vesicles.^68^^,^^69^ Furthermore, overexpression of PI(4,5)P_2_-producing enzyme PIPKIγ promotes E-cadherin trafficking to the plasma membrane in a kinase-activity-dependent manner.^67^ PI(4,5)P_2_ is primarily localized to the plasma membrane, but it is also present in the Golgi apparatus, where it plays a critical role in the structural organization of the Golgi apparatus.^66^ Depletion of PI(4,5)P_2_ results in the dissociation of βIII-spectrin, a PI(4,5)P_2_-interacting protein, from the Golgi apparatus and fragmentation of the Golgi apparatus in both isolated Golgi membranes and cultured cells.^70^^,^^71^^,^^72^ Consistent with these findings, we showed that treatment with the PIPKIγ inhibitor UNC3230 decreased the PI(4,5)P_2_ level, induced rounding of the Golgi apparatus, and reduced E-cadherin expression at the ventricular surface. Thus, the PI(4,5)P_2_ reduction in the RGCs of *Mboat7* KO mice may impair the intracellular transport of E-cadherin by affecting PI(4,5)P_2_-dependent trafficking process and/or the organization of the Golgi apparatus.

Although changes in fatty acid composition of PI and PIPs were observed in E11.5 cortices of *Mboat7* KO mice, the decrease in the amount of PI(4,5)P_2_ in the cortex of *Mboat7* KO mice became evident from E12.5, suggesting that PI(4,5)P_2_ synthesis is perturbed starting around E12.5. PI(4,5)P_2_ is generated from PI4P by PI4P 5-kinases (PIPKIs). Among the three PIPKI isoforms (α, β, γ), PIPKIγ is the dominant isoform in the brain since PIPKIγ KO mice show a decreased amount of PI(4,5)P_2_ in the brain while PIPKIα KO, PIPKIβ KO, and PIPKIα/β double KO mice do not.^73^ Expression of PIPKIγ in the brain is detected at E12 and increases with embryonic development.^73^ Consistently, our lipidomic analyses revealed that the total amounts of PI, PI4P, and PI(4,5)P_2_ increased from E11.5 through E13.5, suggesting that both PIPKIγ and its substrates rise in parallel to promote PI(4,5)P_2_ production. PIPKIs have been reported to prefer arachidonic-acid-containing PI4P as a substrate over saturated-fatty-acid-containing PI4P.^74^^,^^75^ Interestingly, PIPKIs possess an arachidonic acid-recognition motif, which is conserved among the enzymes with specificity for arachidonic acid, such as diacylglycerol kinase epsilon and lipoxygenases.^75^ In contrast, such acyl chain selectivity and the recognition motif are not found in other PI/PIP kinases.^76^ Consistent with the arachidonic acid preference of PIPKIs, UNC3230 selectively reduced PI(4,5)P_2_ species with arachidonic acid in the hemisphere cultures. Therefore, PIPKIγ, whose expression in the brain starts at E12, may fail to produce a sufficient amount of PI(4,5)P_2_ in the *Mboat7* KO cortex, where the level of PI4P with arachidonic acid is reduced. Considering that UNC3230 phenocopies *Mboat7* deficiency, the reduction of arachidonic acid in PI and PI4P may primarily affect the production and levels of PI(4,5)P_2_, while it may exert only a minor influence on the intrinsic functions of PI and PI4P themselves.

Taken together, these results strongly suggest that reduced PI(4,5)P_2_ levels resulting from impaired PI remodeling disrupt RGC integrity, ultimately leading to microcephaly in *Mboat7* deficiency. Our findings provide mechanistic insights not only into microcephaly associated with *MBOAT7* deficiency but also into the importance of arachidonic acid in embryonic brain development. These findings suggest some potential therapeutic strategies for treating human neurodevelopmental disorders arising from *MBOAT7* mutations, such as supplementation with arachidonic acid or arachidonic-acid-containing PI and pharmacological activation of PIPKIγ.

### Limitations of the study

A limitation of the current study is that our analyses focused primarily on RGCs, and it remains unclear whether the observed molecular and cellular defects also occur in other cell types in the developing neocortex. For instance, PI(4,5)P_2_ levels might also be reduced in IPCs or postmitotic neurons, but the consequences of such reductions have not been fully addressed in this study. Since IPCs and neurons do not contact the ventricular surface, any functional impairments in these cells would likely differ from the RGC defects observed in this study. Nevertheless, it is possible that reduced PI(4,5)P_2_ in IPCs could impair their differentiation into neurons, which would contribute to a reduction in neuronal numbers in the *Mboat7* KO cortex. Further studies will be required to elucidate the effects of reduced PI(4,5)P_2_ in other neural cell types.

## Resource availability

### Lead contact

Further information and requests for resources and reagents should be directed to and will be fulfilled by the lead contact, Nozomu Kono (nozomu@mol.f.u-tokyo.ac.jp).

### Materials availability

This study did not generate new unique reagents.

### Data and code availability

• All data reported in this paper will be shared by the lead contact upon request.
• RNA sequencing data have been deposited at the GEO repository and are publicly available, with the accession number GSE288356. Lipidomic data have been deposited at Metabolomics Workbench and are publicly available, with the Study ID ST003747, ST003748, and ST003749.
• This paper does not report original code.
• Any additional information required to reanalyze the data reported in this work paper is available from the lead contact upon request. A preprint version of this manuscript was previously posted on bioRχiv (https://doi.org/10.1101/2024.04.30.588048).

## Acknowledgments

We thank S. Nishikawa (RIKEN) for providing *Sox1-Cre* mice and Y. Nakamura (Tokyo University of Science) and K. Kanemaru (Tokyo University of Science) for helpful discussions. Public access to metabolomics data via Metabolomics Workbench is supported by NIH grants U2C-DK119886 and OT2-OD030544. This work was supported by grants from the 10.13039/501100001691Japan Society for the Promotion of Science 10.13039/501100001691KAKENHI (grant numbers 23K14343 [to Y.I.] and 17H06164 and 17H06418 [to H.A.]); the 10.13039/100009619Japan Agency for Medical Research and Development (grant numbers JP21gm1210013 [to N.K.] and JP21gm0010004h9905 and JP22ck0106533h0003 [to J.A.]); 10.13039/501100002241Japan Science and Technology Agency 10.13039/501100020963Moonshot R&D Program (grant numbers JPMJMS2023-11 [to J.A.] and JPMJMS2023-15 [to J.A.]).

## Author contributions

Y.I., conceptualization, formal analysis, investigation, methodology, and writing—original draft. Y.K., investigation, methodology, and writing—review & editing. T.I., investigation and methodology. N.K., methodology and resources and writing—review & editing. H.A., funding acquisition, supervision, and writing—review & editing. Y.G., resources, supervision, and writing—review & editing. J.A., funding acquisition, supervision, and writing—review & editing. N.K., conceptualization, formal analysis, funding acquisition, supervision, and writing—original draft.

## Declaration of interests

The authors declare no competing interests.

## STAR★Methods

### Key resources table

**Table:** 

REAGENT or RESOURCE	SOURCE	IDENTIFIER
**Antibodies**		

Rabbit anti-cleaved caspase-3	Cell Signaling	Cat# 9661; RRID: AB_2341188
Rabbit anti-caspase-3	Cell Signaling	Cat# 9662S; RRID: AB_331439
Rabbit anti-Sox2	Cell Signaling	Cat# 3728S; RRID: AB_2194037
Mouse anti-phospho-Histone H3	Cell Signaling	Cat# 9706S; RRID: AB_331748
Mouse anti-Sox2	Santa Cruz Biotechnology	Cat# sc-365964; RRID: AB_10843364
Goat anti-Sox2	Santa Cruz Biotechnology	Cat# sc-17320; RRID: AB_2286684
Rabbit anti-Tbr1	Abcam	Cat# Ab31940; RRID: AB_2200219
Rat anti-Ctip2	Abcam	Cat# Ab18465; RRID: AB_2064130
Rabbit anti-Tbr2	Abcam	Cat# Ab23345; RRID: AB_778267
Mouse anti-PCNA	Abcam	Cat# Ab29; RRID: AB_303394
Rat anti-BrdU	Abcam	Cat# Ab6326; RRID: AB_305426
Rat anti-Tbr2	Thermo Fisher Scientific	Cat# 14-4875-80; RRID: AB_11043546
Rat anti-Cux1	Novus Biologicals	Cat# NBP2-13883; RRID: AB_3261458
Mouse anti-PI(4,5)P_2_	Echelon Biosciences	Cat# Z-P045; RRID: AB_427225
Rabbit anti-ZO-1	Zymed Laboratories	Cat# 617300; RRID: AB_2533938
Mouse anti-Aurora B	BD Transduction Laboratories	Cat# 611082; RRID: AB_2227708
Moue anti-BrdU	BD Transduction Laboratories	Cat# 555627; RRID: AB_395993
Mouse anti-E-cadherin	BD Transduction Laboratories	Cat# 610181; RRID: AB_397580
Mouse anti-N-cadherin	BD Transduction Laboratories	Cat# 610920; RRID: AB_2077527
Mouse anti-Nestin	BD Transduction Laboratories	Cat# 611658; RRID:AB_399176
Mouse anti-GM130	BD Transduction Laboratories	Cat# 610822; RRID: AB_398141
Mouse anti-GAPDH	Merck Millipore	Cat# 6C5; RRID: AB_2858176
Mouse anti-GAPDH	Proteintech	Cat# 60004-1-Ig; RRID: AB_2107436
Rabbit anti-Pax6	Merck Millipore	Cat# AB2237; RRID: AB_1587367
Chicken anti-Tbr2	Merck Millipore	Cat# AB15894; RRID: AB_10615604
Rabbit anti-Cux1	Proteintech	Cat# 11733-1-AP; RRID: AB_2086995
Rat anti-LPLAT11	Our laboratory	N/A
Donkey anti-mouse IgG, Alexa 488-conjugated	Thermo Fisher Scientific	Cat# A21202; RRID: AB_141607
Donkey anti-mouse IgG, Alexa 555-conjugated	Thermo Fisher Scientific	Cat# A31570; RRID: AB_2536180
Donkey anti-mouse IgG, Alexa 647-conjugated	Thermo Fisher Scientific	Cat# A31571; RRID: AB_162542
Donkey anti-mouse IgM, Alexa 488-conjugated	Thermo Fisher Scientific	Cat# A21042; RRID: AB_141357
Donkey anti-rabbit IgG, Alexa 488-conjugated	Thermo Fisher Scientific	Cat# A21206; RRID: AB_2535792
Donkey anti-rabbit IgG, Alexa 647-conjugated	Thermo Fisher Scientific	Cat# A31573; RRID: AB_2536183
Donkey anti-rat IgG, Alexa 488-conjugated	Thermo Fisher Scientific	Cat# A21208; RRID: AB_2535794
Donkey anti-rat IgG, Alexa 555-conjugated	Thermo Fisher Scientific	Cat# A78945; RRID: AB_2910652
Donkey anti-goat IgG, Alexa 488-conjugated	Thermo Fisher Scientific	Cat# A11055; RRID: AB_2534102
Donkey anti-goat IgG, Alexa 647-conjugated	Thermo Fisher Scientific	Cat# A21447; RRID: AB_2535864
Donkey anti-Chicken IgY, Alexa 555-conjugated	Thermo Fisher Scientific	Cat# A78949; RRID: AB_2921071
Anti-Mouse IgG, HRP-Linked F(ab’)2 Fragment, Sheep	Cytiva	Cat# NA9310V; RRID: AB_772193
Anti-Rabbit IgG, HRP-Linked F(ab’)2 Fragment, Donkey	Cytiva	Cat# NA9340V; RRID: AB_772191
Anti-Rat IgG, HRP-Linked F(ab’)2 Fragment, Goat	Cosmobio	Cat# A103PT

**Chemicals, peptides, and recombinant proteins**		

RNeasy Plus Mini Kit	QIAGEN	Cat# 74134
KOD One	TOYOBO	Cat# KMM-101
4′,6-Diamidino-2-phenylindole dihydrochloride	Sigma-Aldrich	Cat# D9542
BrdU	Sigma-Aldrich	Cat# B5002
IdU	Tokyo Chemical Industry Co.,Ltd.	Cat# I0258
Sevenin-1	Enamine Ltd.	Cat# Z1084980652
UNC3230	TargetMol	Cat# T23498
17:0-20:4 PI	Avanti Polar Lipids	Cat# LM1502
17:0-20:4 PI4P	Avanti Polar Lipids	Cat# LM1901
17:0-20:4 PI(4,5)P_2_	Avanti Polar Lipids	Cat# LM1904
12:0-13:0 PI	Avanti Polar Lipids	Cat# LM1500
12:0-13:0 PS	Avanti Polar Lipids	Cat# LM1300
12:0-13:0 PC	Avanti Polar Lipids	Cat# LM1000
12:0-13:0 PE	Avanti Polar Lipids	Cat# LM1100
(Trimethylsilyl)diazomethane	Sigma-Aldrich	Cat# 362832
Acetic Acid for LC/MS	Fujifilm	Cat# 018-20061

**Deposited data**		

Bulk RNA-seq data	Gene Expression Omnibus (GEO) database	GSE288356 ID: 200288356
LC-MS/MS data	Metabolomics workbench	ID: ST003747
SFC-MS/MS data	Metabolomics workbench	ID: ST003748, ST003749
Arachidonic acid incorporation into phosphatidylinositol by LPLAT11/MBOAT7 ensures radial glial cell integrity in developing neocortex	BioRχiv	https://doi.org/10.1101/2024.04.30.588048

**Experimental models: Organisms/strains**		

Mouse: C57BL/6N	CLEA Japan	N/A
Mouse: *Mboat7* KO	This laboratory	N/A
Mouse: B6.B6CB-Sox1<tm1(cre)Take>	Riken	No. CDB0525K

**Oligonucleotides**		

Primers to amplify mClover3 for cloning into the pCAGGS vector forward: ATCCACCGGCCG GTCGCCACCATGGTGAGCAAGGGCGAGGA; reverse: CTTCTGCTCCTCGAGCTACTTGT ACAGCTCGTCCATGC	Eurofins Genomics	N/A
Primers to amplify E-cadherin for cloning into the pCAGGS vector forward: CCGGGTACCGAATT CGCCACCATGGGAGCCCGGTGCCGCAG reverse: CTTCTGCTCCTCGAGCTAGTCGT CCTCACCACCGCCGTAC	Eurofins Genomics	N/A

**Software and algorithms**		

Prism (version 8)	GraphPad Software	https://www.graphpad.com
Fiji/ImageJ	Fiji/ImageJ	https://imagej.net/software/fiji/
Analyst1.7.2	AB SCIEX	https://sciex.jp/products/software/analyst-software
MultiQuant 3.0.3	AB SCIEX	https://sciex.jp/products/software/multiquant-software

**Other**		

Confocal laser microscope	Leica	SP8
Cyostat	Leica	CM1950
Luminescent image analyzer	Cytiva	ImageQuant LAS4000
Western blot and chemiluminescence imaging system	VILBER	Fusion Solo S
Nitrogen gas generator	SYSTEM INSTRUMENTS Co.,Ltd.	Model 05BL
Sonicator	Branson	SFX 250
Microinjector	Eppendorf	Femtojet 4i
Elecroporator	Nepagene	NEPA21
Electrodes	Nepagene	CUY650-P3
Triple quadrupole linear ion trap mass spectrometer	AB SCIEX	QTRAP4500

### Experimental model and study participant details

#### Animals

Because patients with *MBOAT7* null mutations exhibit microcephaly, we used *Mboat7*-deficient mice, which also develop microcephaly, as a model in this study. *Mboat7* KO mice and floxed mice (*Mboat7*^*fl/fl*^) were generated previously.^4^^,^^9^ *Sox1-Cre* mice were provided by RIKEN (No. CDB0525K, https://large.riken.jp/distribution/mutant-list.html). To generate Sox1-KO mice (*Mboat7*^*fl/fl*^*; Sox1-Cre*), *Sox1-Cre* mice were first crossed with *Mboat7*^*fl/fl*^ mice, and *Mboat7*^*+/fl*^*; Sox1-Cre* offspring were then crossed with *Mboat7*^*fl/fl*^ mice. They were maintained in C57BL/6N. All mice were housed in climate-controlled (23 °C) pathogen-free facilities with a 12 h light-dark cycle, with free access to standard chow (CE2; CLEA Japan) and water. All animal experiments were performed in accordance with protocols approved by the Animal Committees of the University of Tokyo in accordance with the Standards Relating to the Care and Management of Experimental Animals in Japan (Approval number: PH3-5 (until 2024) and A2024P008 (since 2025)). For timed pregnancy, mice were mated at 7:00 p.m. and checked for vaginal plug at 10:00 a.m. next morning. The timing when detection of the vaginal plug was designated as E0.5. All embryos (E10.5-E18.5), pups (P5), and 3-week-old mice were collected regardless of gender because *Mboat7* KO mice exhibit microcephaly in both sexes and were randomly assigned to experimental groups.

### Method details

#### BrdU labeling

For BrdU labeling, E12.5 or E13.5 pregnant mice were given an intraperitoneal injection of 50 mg/kg BrdU. To investigate differentiation of RGCs into neurons or IPCs, BrdU injections were given 24 h before sacrifice. To label cells in the S/G2 phase, early G1 phase, and late G1 phase in *Mboat7* KO mice, mice were injected with BrdU 2 h, 8.5 h, and 17 h before harvesting, based on the cell cycle length.

#### Cell cycle length

For measuring S-phase length and total cell length, E12.5 or E13.5 pregnant mice were given an intraperitoneal injection of 50 mg/kg IdU, followed by an injection of BrdU 1.5 h later. Embryos were harvested 0.5 h after BrdU injection. Cell cycle lengths in RGCs and IPCs were calculated using the following paradigm^77^^,^^78^: Cells in the leaving fraction (*L*_*cells*_) were identified as those labeled with the IdU antibody (BD555627), which recognizes both IdU and BrdU, but not the BrdU antibody (Ab6326), which specifically recognizes BrdU. The population of cells labeled with BrdU is designated as *S*_*cells*_. The lengths of S-phase (Ts) and of the entire cell cycle (Tc) were calculated using the following formula:

$$T_{S}=S_{cells}/L_{cells}\times 1.5$$

$$T_{C}=P_{cells}/L_{cells}\times 1.5-2$$

where *P*_*cells*_ is the total number of RGCs (Sox2^+^) in the ventricular zone or of IPCs (Tbr2^+^) in the subventricular zone.

#### Immunohistochemistry

For immunofluorescence staining, embryonic brains were postfixed with ice-cold 4% (wt/vol) paraformaldehyde (PFA) at 4 °C for 2–3 h. Postnatal brains were post-fixed overnight at 4 °C. The brains were then equilibrated with 15% (w/v) sucrose in PBS at 4 °C overnight, followed by 30% (w/v) sucrose in PBS at 4 °C overnight, and finally frozen in OCT (Tissue TEK). Coronal sections (14 μm thick) were permeabilized with TBST buffer (25 mM Tris-HCl, pH 7.5, 140 mM NaCl, 0.1% Triton X-100) for 30 min, blocked with 3% BSA-TBST for 1 h at room temperature, and incubated overnight at 4 °C with primary antibodies in blocking buffer. For staining with the antibodies to Cux1, Ctip2, Tbr1, Tbr2, Sox2 (sc-365964, sc-17320), p-H3, PCNA, IdU, BrdU, and Aurora B, we performed antigen retrieval by autoclave treatment of sections with 10 mM citrate buffer (pH 6.0) for 10 min at 105 °C. For staining with the antibody to PI(4,5)P_2_, TBS containing 0.01% saponin was used for permeabilization, and 3% BSA-TBS containing 0.01% saponin was used for blocking. Sections were incubated with Alexa-Fluor-labeled secondary antibodies and DAPI in a blocking buffer for 2 h at room temperature. Fluorescence images were obtained with a laser confocal microscope (Leica TCS-SP5 and SP8). Alexa-Fluor-labeled secondary antibodies and DAPI were obtained from Sigma-Aldrich.

#### Measuring the length of the Golgi apparatus

To measure the length of the Golgi apparatus of the cortices, frozen sections were stained for GM130, and all GM130^+^ Golgi apparatus in the ventricular zone within 50-μm-wide bins were measured manually using ImageJ. For each embryo, the average length of all measured Golgi apparatus was calculated and analyzed as an independent data point.

#### *Ex vivo* culture of cortical hemispheres

*Ex vivo* culture of cortical hemispheres was performed as described previously^46^ with some modifications. Briefly, the brains of embryos were dissected, and two cerebral cortical hemispheres were separated in individual 35 mm dishes containing Dulbecco’s Modified Eagle Medium (DMEM) with 1% penicillin/streptomycin/glutamine. The separated hemispheres were then transferred to a new 35 mm dish containing culture medium (Opti-MEM I with 20 mM glucose, 55 μM 2-mercaptoethanol, and 1% penicillin/streptomycin/glutamine) using a P1000 Pipetman with a cut pipette tip and kept on ice until all dissections were completed. The hemispheres were subsequently transferred to individual wells of a 12-well plate containing 2 mL of culture medium. The plates were then placed on a nutator inside a 5% CO_2_ incubator at 37 °C for 24 h.

For LPLAT11 inhibitor treatment, N-[2-(2-bicyclo[2.2.1]heptanyl)ethyl]-4-[2-(methylsulfamoyl)phenyl]piperazine-1-carboxamide (Sevenin-1; Enamine Company),^14^ dissolved in DMSO, was added to the culture medium at a final concentration of 10 μM, which did not exhibit any apparent toxicity, whereas 50 μM caused marked toxicity, such as collapse of hemispheres. For PIPKIγ inhibitor treatment, UNC3230 (TargetMol Chemicals Inc.) dissolved in DMSO was added to the culture medium at a final concentration of 1 μM, which reduced the level of PI(4,5)P_2_ without any apparent toxicity among the tested concentrations (0.25, 0.5, and 1 μM).

#### Inhibitor injection into lateral ventricle

For PIPKIγ inhibition, UNC3230 was diluted to 4 μM in PBS, mixed with the same amount of CFSE containing fastgreen, and injected into the lateral ventricle of each littermate at E12.5. PBS containing 0.1% DMSO was used as a control. The uterine horn was placed back into the abdominal cavity to allow embryos to continue development. The next day, the embryos were collected for immunohistochemical analysis of the brain.

#### *In utero* electroporation

Introduction of plasmid DNA into the neuroepithelial cells of mouse embryo *in utero* was performed as described.^79^ At E12.5, 1–2 μL of a plasmid mixture containing pCAGGS-mClover3 (1 μg/μL) and either pCAGGS-empty vector (2 μg/μL) or pCAGGS-mouse E-cadherin (2 μg/μL) was injected into the lateral ventricle of each littermate. Electrodes were placed flanking the equivalent ventricular region of each embryo, covered with a drop of PBS, and pulsed 4 times at 30 V for 30 ms, separated by intervals of 970 ms with an electroporator (NEPA21; NEPA Gene). The uterine horn was placed back into the abdominal cavity to allow the embryo to continue development. The next day, the embryos were collected for immunohistochemical analysis of the brain.

#### Western blotting

Cortices from E12.5 to E18.5 were isolated in ice-cold PBS including 8 mM NaF, 12 mM beta-glycerophosphate, 1 mM Na_3_VO_4_, 1.2 mM Na_2_MoO_4_, 5 μM Cantharidin, and 2 mM Imidazole and collected in lysis buffer (62.5 mM Tris-HCl pH 6.8, 10% Glycerol, 1% SDS) and sonicated. The lysate was incubated at room temperature for 30 min and then centrifuged at 15,000 g for 30 min at room temperature, and the supernatants were collected. The protein concentration was determined by the BCA assay (Pierce). Proteins were separated by SDS-PAGE and transferred to PVDF membranes (Millipore). The membranes were blocked with 5% skimmed milk in TTBS buffer (10 mM Tris-HCl, pH 7.4, 150 mM NaCl, 0.05% Tween 20) for 1 h at room temperature and then incubated with primary antibodies overnight at 4°C. On the next day, the membranes were incubated with horseradish peroxidase-conjugated anti-mouse, anti-rabbit, or anti-rat antibody. Proteins were detected by enhanced chemiluminescence (ECL western blotting detection system, Cytiva) using the ImageQuant LAS 4000 system (Cytiva) and Fusion Solo S (VILBER).

#### Lipid extraction

Lipid extractions were conducted by the method of Bligh and Dyer.^80^ The extracted solutions were dried up with a centrifugal evaporator, dissolved in chloroform/methanol (2/1, v/v), and stored at −20°C. The phospholipid content of samples was determined by phosphorus assay. Samples were dried up under a stream of nitrogen at room temperature and dissolved in a 1:1 mixture of isopropanol and methanol containing 0.25 μM internal standards (12:0/13:0 PC, PE, PS, PI) was added to achieve a final concentration of 300 μM phospholipids.

For preparation of SFC-MS samples, cortices from E11.5 to E13.5 were isolated in ice-cold DMEM, washed with ice-cold 1 M HCl, and immediately transferred into 2 mL safe-lock poly-propylene tubes containing 750 μL of ice-cold quench mix, followed by sonication. Then, 170 μL of H_2_O was added, and the samples were stored at −80°C prior to lipid extraction. Lipid extraction was performed according to the procedures by Clark et al*.*^81^ The single-phase sample consisting of 170 μL of an aqueous sample, 10 ng of internal standards [17:0/20:4 PI, 17:0/20:4 PI4P, and 17:0/20:4 PI(4,5)P_2_], and 750 μL of quench mix, was mixed with 725 μL of CHCl_3_ and 170 μL of 2 M HCl. The mixture was then vortexed and centrifuged (1,500 g, 5 min at room temperature). The lower organic phase was collected into a new 2 mL safe-lock poly-propylene tube, mixed with 708 μL of pre-derivatization wash solution, vortex-mixed, and centrifuged (1,500 g, 3 min at room temperature). The lower phase was collected into another fresh tube and subjected to derivatization.

#### Derivatization of extracted lipids

Derivatization of lipids was performed in a fume hood with appropriate personal safety equipment, following the procedures described by Clark et al*.*^81^ Fifty μL trimethylsilyl diazomethane in hexane (2 M solution; Sigma-Aldrich) was added to the lipid extracts (∼1 mL), and the reaction was allowed to stand for 10 min at room temperature. The reaction was quenched with 6 μL of acetic acid (LC-MS grade; Fujifilm). Subsequently, 700 μL post-derivatization wash solution was added, and the mixture was centrifuged (1,500 g, 3 min). The lower phase was collected and rewashed with a 700 μL post-derivatization wash solution. The samples were temporarily stored at −80°C. Ninety μL of methanol and 10 μL of H_2_O were added to the final collected lower phase. The samples were then dried up using a centrifugal evaporator. After dissolving in 80 μL of methanol, the samples were sonicated for 1 min, followed by the addition of 20 μL of H_2_O. Following centrifugation at 15,000 g for 30 min at 4 °C, the supernatants were collected. To avoid degradation, the samples were stored at −80°C until use.

#### LC-MS analysis of phospholipids

LC/ESI-MS–based lipidomic analyses were performed on a Shimadzu Nexera UPLC system (Shimadzu) coupled with a QTRAP 4500 hybrid triple quadrupole linear ion trap mass spectrometer (SCIEX). Chromatographic separation was performed on a SeQuant ZIC-HILIC PEEK coated column (250 mm × 2.1 mm, 1.8 μm; Millipore) maintained at 50 °C using mobile phase A (water/acetonitrile (95/5, v/v) containing 10 mM ammonium acetate) and mobile phase B (water/acetonitrile (50/50, v/v) containing 20 mM ammonium acetate) in a gradient program (0–22 min: 0% B→40% B; 22–25 min: 40% B→40% B; 25–30 min: 0% B) with a flow rate of 0.3 mL/min. The specific detection of phospholipid species was performed using MRM, as described in Table S2. Analyst (SCIEX) was used for data acquisition and processing. MultiQuant (SCIEX) was used for data evaluation. Gaussian smoothing width was 2.0 points. The peak area of the lipid species was divided by the corresponding internal standard area, and the total amount of phospholipids per cortex or hemisphere was calculated from the ratio of injection volume to total sample volume.

#### SFC-MS analysis of PIPs

For the detection of PIPs, SFC-MS/MS-based lipidomic analyses were performed on a Shimadzu Nexera UC/s system (Shimadzu) coupled with a QTRAP 4500 hybrid triple quadrupole linear ion trap mass spectrometer (SCIEX). Lipids extracted were injected by an autosampler; typically, 10 μL of the sample was applied. Chromatographic separation was performed on an ULTRON AF-HILIC-CD (250 mm × 2.1 mm, 5.0 μm; Shinwa Chemical Industries) maintained at 10 °C using immersion cooler *Neo* cool drip BE201F (Yamato Scientific) with mobile phase A [supercritical carbon dioxide (SCCO_2_)] and mobile phase B [water/methanol (2.5/97.5, v/v) containing 0.1% (v/v) formic acid] in a gradient program (0–16 min: 5% B→20% B; 16.01–18 min: 40% B; 18.01–22 min: 5% B) with a flow rate of 1.5 mL/min. The instrument parameters of QTRAP4500 for positive ion mode were as follows: curtain gas, 10 psi; ionspray voltage, 4500 V; temperature, 500 °C; CAD, 7 (arbitrary unit); ion source gas 1, 30 psi; ion source gas 2, 70 psi. The specific detection of PI and PIP species was performed using MRM, as described in Table S3. Analyst (SCIEX) was used for data acquisition and processing. MultiQuant (SCIEX) was used for data evaluation. Gaussian smoothing width was 2.0 points. Retention time of PI4P and PI(4,5)P_2_ is confirmed by measuring internal standards [17:0/20:4 PI3P, PI4P, PI5P, PI(3,4)P_2_, PI(3,5)P_2_, and PI(4,5)P_2_] before measuring samples. Peak areas of PI4P and PI(4,5)P_2_ were integrated from the ion chromatogram of PIP and of PIP_2_, respectively, based on retention time,^45^ and isotopic correction was applied. The peak area of the lipid species was divided by the corresponding internal standard area, and the total amount of PIPs per cortex or hemisphere was calculated from the ratio of injection volume to total sample volume.

#### MS imaging of PI and PIPs

E13.5 brains were embedded in 2% carboxymethylcellulose and frozen in liquid nitrogen. The brain sections (10 μm thick) were washed by submerging in 50 mM of ammonium formate for 5 s. MALDI matrix *p*-NA was applied to sections as previously described.^82^ Briefly, *p*-NA was dissolved in 80% EtOH containing 150 mM ammonium formate. The section was sprayed with a SunCollect automated sprayer at an air pressure of 0.3 MPa and a 15 μL/min flow rate. First, the brain sections were sprayed with 10 layers of 1 mg/mL *p*-NA, and then 25 layers of 10 mg/mL *p*-NA. MALDI-MSI analyses were performed using Fourier transform orbital trapping MS (QExactive, Thermo Fisher Scientific, San Jose, CA) connected to a MALDI laser system (AP-SMALDI5, TransMIT, Giessen, Germany) in full-scan mode at a mass resolution of 140,000. The laser was manually focused. The laser power was set at a 27° attenuator setting to yield optimal results. Ion images were reconstructed using IMAGEREVEAL MS (Shimadzu, Kyoto, Japan). For data presentation, the m/z signals corresponding to PI and PIPs molecular species were extracted and normalized to the total ion current (TIC).

#### RNA-seq

E11.5 and E12.5 cortices were dissected in ice-cold DMEM, and total RNA was isolated with RNeasy Mini Kit (Qiagen) and RNase-Free DNase Set (79254, Qiagen) following the manufacturer’s instructions. Sequencing of E11.5 cortex was performed at Rhelixa, Inc., Japan, using the Illumina NovaSeq 6000 platform. Sequencing of E12.5 cortex was performed at KOTAI Biotechnologies, Inc., Japan, using the Illumina NovaSeq X Plus platform. Because the data for E11.5 and E12.5 cortices were obtained from different providers and platforms, direct comparison between these stages would require batch effect correction to account for potential technical differences.

### Quantification and statistical analysis

Statistical analyses were performed using GraphPad Prism 8 software. The sample size was indicated in the figure legends. Data were presented as means ± SEM. F-test, followed by the unpaired two-tailed Student’s *t* test or Welch’s *t* test, was applied to determine significant differences between the two samples. For comparison of more than two groups, data were compared using one-way ANOVA followed by Tukey’s test. Differences were considered significant for *p*-values <0.05 (∗*p* < 0.05, ∗∗*p* < 0.01, ∗∗∗*p* < 0.001). Blinding was performed by analyzing the samples without knowing the genotypes.

### Additional resources

A preprint version of this manuscript was previously posted on bioRχiv (https://www.biorxiv.org/content/10.1101/2024.04.30.588048v1).
