# Supplementary material for: LPLAT11/MBOAT7-driven phosphatidylinositol remodeling ensures radial glial cell integrity in developing neocortex
Source: iScience. 2025 Nov 27;29(1):114248. doi: 10.1016/j.isci.2025.114248 (PMC12757648; doi:10.1016/j.isci.2025.114248)

iScience, Volume *29*

Supplemental information

LPLAT11/MBOAT7-driven phosphatidylinositol remodeling ensures radial glial cell integrity in developing neocortex

Yuki Ishino, Yusuke Kishi, Taiga Iwama, Naohiro Kuwayama, Hiroyuki Arai, Yukiko Gotoh, Junken Aoki, and Nozomu Kono

Supplemental Figures

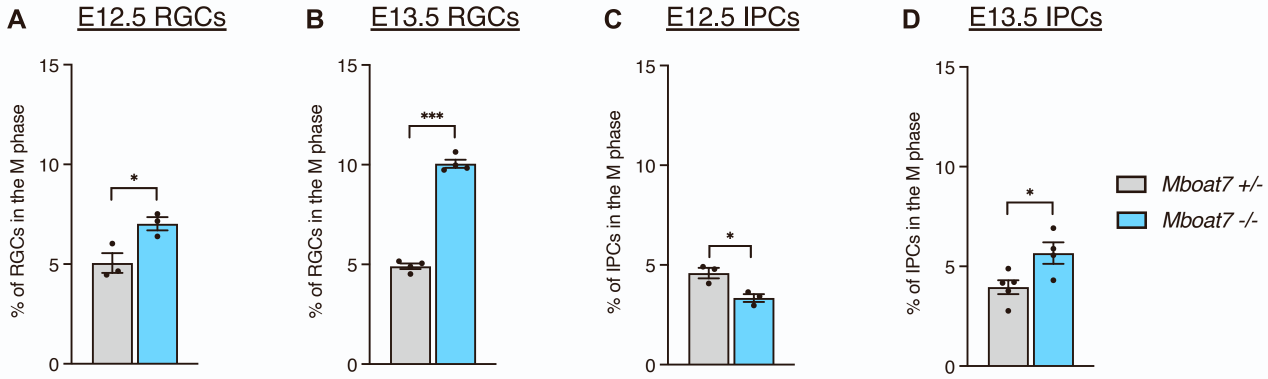

Figure S1. M-phase duration is prolonged in E12.5 and E13.5 *Mboat7* KO RGCs, related to Figure 3. (A,B) Population of RGCs in the M phase in *Mboat7*$^{+/-}$ and *Mboat7*$^{-/-}$ mice at E12.5 (A) and E13.5 (B) (n=3 embryos (A) and n=4 embryos (B) from two independent litters for each genotype). (C,D) Population of IPCs in the M phase in *Mboat7*$^{+/-}$ and *Mboat7*$^{-/-}$ mice at E12.5 (C) and E13.5 (D) (n=3 embryos (E12.5), n=5 embryos (E13.5 *Mboat7*$^{+/-}$), and n=4 embryos (E13.5 *Mboat7*$^{-/-}$) from two independent litters). Data are shown as mean ± SEM; *$p < 0.05$, ***$p < 0.001$; unpaired two-tailed Student's t-test.

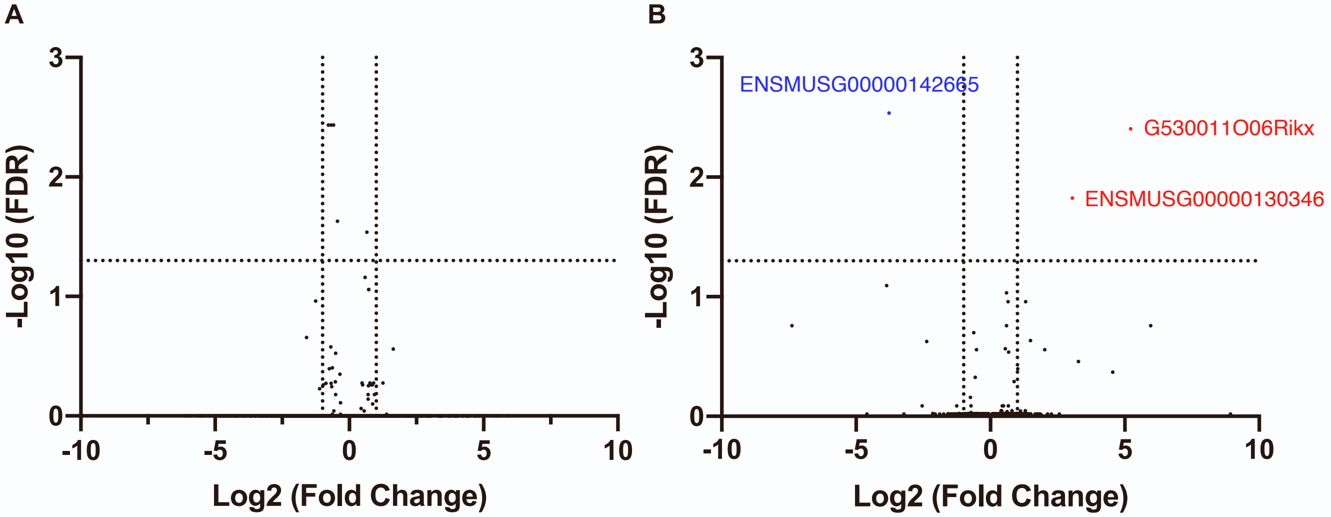

Figure S2. RNA-seq analysis of E11.5 and E12.5 cortices, related to Figure 6.

Volcano plot depicting the results of the RNA-seq analysis of E11.5 (A) and E12.5 cortices (B) (n=2 embryos for each genotype). Dotted lines show log2 fold change (*Mboat7*$^{-/-}$/*Mboat7*$^{+/-}$) -1, 1, respectively. Red dots and blue dot show statistically significantly upregulated and downregulated genes ($|\log_2 FC| \geqq 1$ and FDR < 0.05) in *Mboat7*$^{-/-}$ mice, respectively.

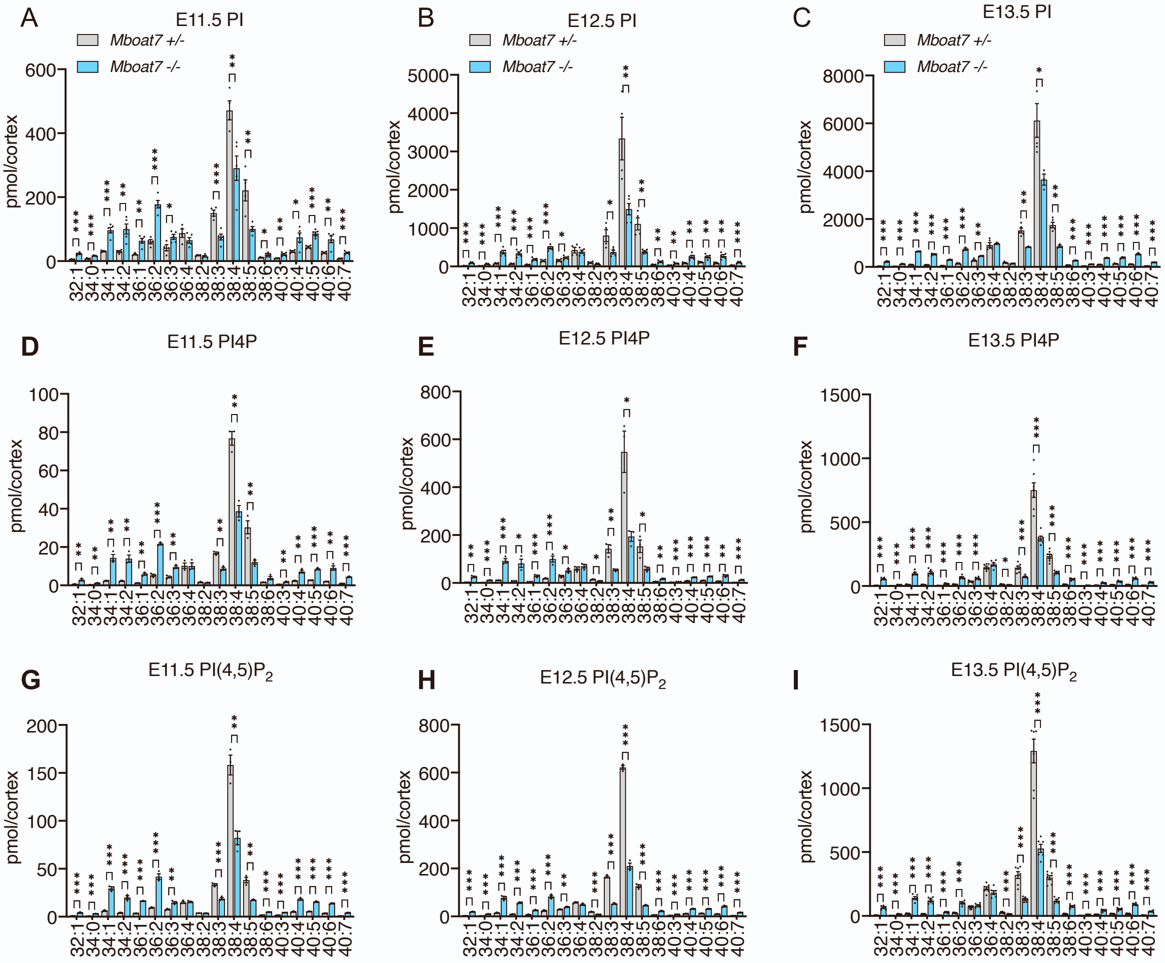

Figure S3. Molecular species of PI, PI4P, and PI(4,5)P_2 in the cortices of E11.5-E13.5 of *Mboat7* KO mice, related to Figure 7.

(A-C) LC-MS/MS analysis of PI at E11.5 (A), E12.5 (B), and E13.5 (C) cortices of *Mboat7*$^{+/-}$ and *Mboat7*$^{-/-}$ mice ((A,B) n=4 embryos (*Mboat7*$^{+/-}$) and n=5 embryos (*Mboat7*$^{-/-}$) from two independent litters for each genotype, (C) n=5 embryos (*Mboat7*$^{+/-}$) and n=3 embryos (*Mboat7*$^{-/-}$) from two independent litters). Peak areas are normalized by the area of the internal standard (25:0 PI). (D-F) SFC-MS/MS analysis of PI4P molecular species at E11.5 (D), E12.5 (E), and E13.5 (F) cortices of *Mboat7*$^{+/-}$ and *Mboat7*$^{-/-}$ mice ((D,E) n=3 embryos from two independent litters for each genotype, (F) n=7 embryos (*Mboat7*$^{+/-}$) and n=6 embryos (*Mboat7*$^{-/-}$) from two independent litters). Peak areas are normalized by the area of the internal standard (37:4 PI4P). (G-I) SFC-MS/MS analysis of PI(4,5)P_2 molecular species at E11.5 (G), E12.5 (H), and E13.5 (I) cortices of *Mboat7*$^{+/-}$ and *Mboat7*$^{-/-}$ mice ((G,H) n=3 embryos from two independent litters for each genotype, (I) n=7 embryos (*Mboat7*$^{+/-}$) and n=6 embryos (*Mboat7*$^{-/-}$) from two independent litters). Peak areas are normalized by the area of the internal standard (37:4 PI(4,5)P_2). Data are shown as mean ± SEM; *$p < 0.05$, **$p < 0.01$, ***$p < 0.001$; multiple *t*-tests.

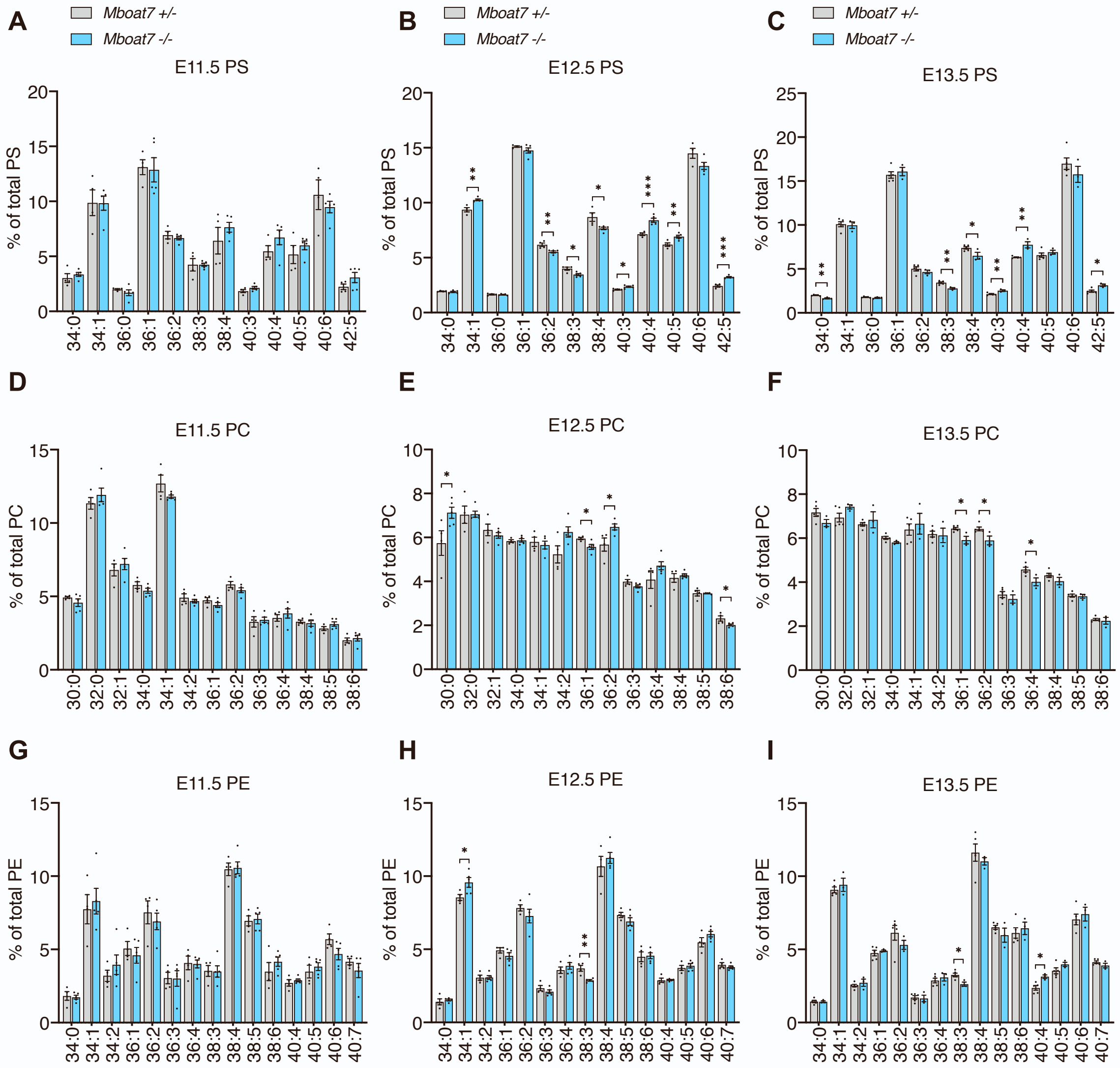

Figure S4. LC-MS/MS analysis of phospholipid species in the cortices of E11.5-E13.5 *Mboat7* KO mice, related to Figure 7.

(A-C) LC-MS/MS analysis of PS at E11.5 (A), E12.5 (B), and E13.5 (C) cortices of *Mboat7*$^{+/-}$ and *Mboat7*$^{-/-}$ mice ((A,B) n=4 embryos (*Mboat7*$^{+/-}$) and n=5 embryos (*Mboat7*$^{-/-}$) from two independent litters, (C) n=5 embryos (*Mboat7*$^{+/-}$) and n=3 embryos (*Mboat7*$^{-/-}$) from two independent litters). Peak areas are normalized by the area of the internal standard (25:0 PS). (D-F) LC-MS/MS analysis of PC at E11.5 (D), E12.5 (E) and E13.5 (F) cortices of *Mboat7*$^{+/-}$ and *Mboat7*$^{-/-}$ mice ((D,E) n=4 embryos (*Mboat7*$^{+/-}$) and n=5 embryos (*Mboat7*$^{-/-}$) from two independent litters, (F) n=5 embryos (*Mboat7*$^{+/-}$) and n=3 embryos (*Mboat7*$^{-/-}$) from two independent litters). Peak areas are normalized by the area of the internal standard (25:0 PC). (G-I) LC-MS/MS analysis of PE at E11.5 (G), E12.5 (H), and E13.5 (I) cortices of *Mboat7*$^{+/-}$ and *Mboat7*$^{-/-}$ mice ((G,H) n=4 embryos (*Mboat7*$^{+/-}$) and n=5 embryos (*Mboat7*$^{-/-}$) from two independent litters, (I) n=5 embryos (*Mboat7*$^{+/-}$) and n=3 embryos (*Mboat7*$^{-/-}$) from two independent litters). Peak areas are normalized by the area of the internal standard (25:0 PE). Data are shown as mean ± SEM; *$p < 0.05$, **$p < 0.01$, ***$p < 0.001$; multiple *t*-tests.

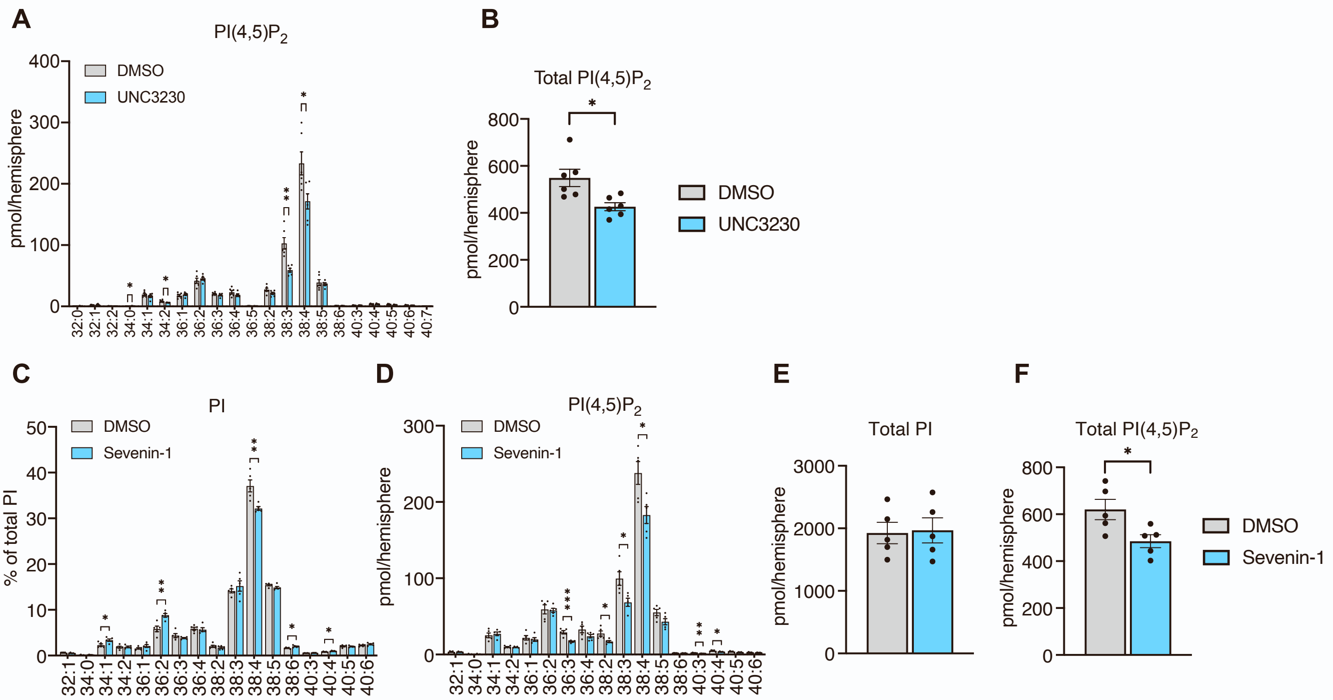

Figure S5. Molecular species and total amounts of PI and PI(4,5)P$_2$ in cultured E12.5 cortical hemispheres treated with UNC3230 or Sevenin-1, related to Figure 7.
(A,B) Molecular species (A) and total amount (B) of PI(4,5)P$_2$ in cultured E12.5 hemispheres treated with 1 μM PIPKIγ inhibitor (UNC3230) (n=6 hemispheres for each group from two independent litters and two independent experiments). Peak areas are normalized by the area of the internal standard (37:4 PI(4,5)P$_2$). (C,D) Molecular species of PI (C) and PI(4,5)P$_2$ (D) in E12.5 hemispheres from wild-type mice cultured in medium with DMSO or 10 μM LPLAT11 inhibitor (Sevenin-1) (n=5 hemispheres for each group from two independent litters and two independent experiments). Peak areas are normalized by the area of the internal standard (37:4 PI or 37:4 PI(4,5)P$_2$). (E,F). Total amounts of PI (E) and PI(4,5)P$_2$ (F) in (C,D) are shown. Data are shown as mean ± SEM; **p* < 0.05, ***p* < 0.01, ****p* < 0.001; unpaired two-tailed Student's *t*-test (B,F) and multiple *t*-tests (A,C,D).

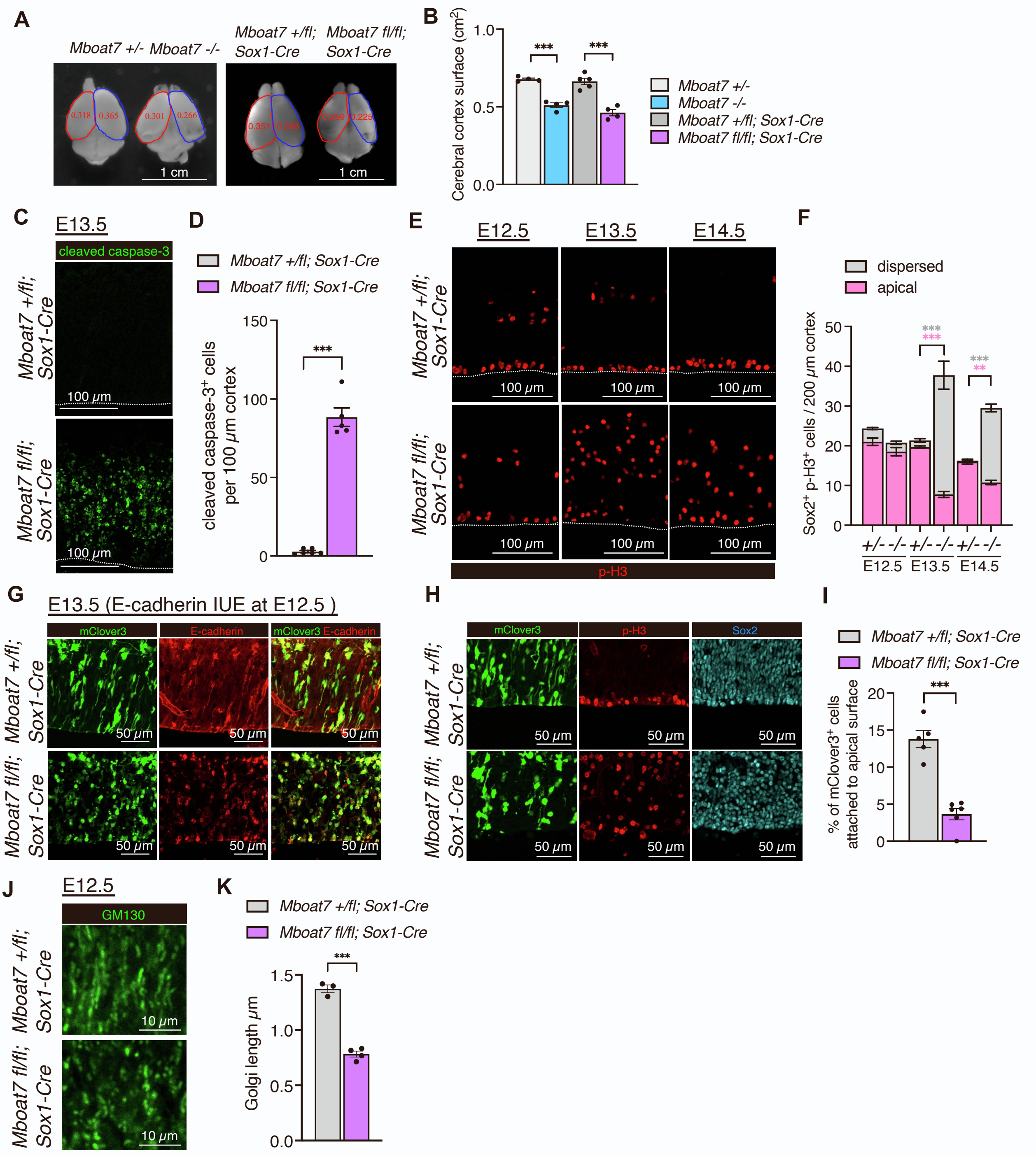

A
Mboat7 +/-
Mboat7 -/-
Mboat7 +/fl; Sox1-Cre
Mboat7 fl/fl; Sox1-Cre
1 cm
B
Cerebral cortex surface (cm^2)
***
Mboat7 +/-
Mboat7 -/-
Mboat7 +/fl; Sox1-Cre
Mboat7 fl/fl; Sox1-Cre
C
E13.5
cleaved caspase-3
100 μm
D
cleaved caspase-3$^+$ cells per 100 μm cortex
E
E12.5
E13.5
E14.5
p-H3
F
dispersed
apical
Sox2$^+$ p-H3$^+$ cells / 200 μm cortex
+/- -/- +/- -/- +/- -/-
E12.5 E13.5 E14.5
G
E13.5 (E-cadherin IUE at E12.5)
mClover3
E-cadherin
mClover3 E-cadherin
50 μm
H
mClover3
p-H3
Sox2
I
% of mClover3$^+$ cells attached to apical surface
J
E12.5
GM130
10 μm
K
Golgi length μm

Figure S6. Neural-specific *Mboat7* KO mice phenocopy global KO mice, related to Figure 7.

(A) Global *Mboat7*$^{+/-}$ and *Mboat7*$^{-/-}$ mice (left), and neural-specific *Mboat7*$^{+/-}$ (*Mboat7*$^{+/fl}$*; Sox1-Cre*) and *Mboat7*$^{-/-}$ mice (*Mboat7*$^{fl/fl}$*; Sox1-Cre*) (right) were sacrificed at 3-week-old. Red and blue lines show the outline of left and right cortices. Areas of cortices (cm^2) are shown in red figures. (B) Total areas of right and left cortices (n=4 mice (*Mboat7*$^{+/-}$ and *Mboat7*$^{-/-}$), n=5 mice (*Mboat7*$^{+/fl}$*; Sox1-Cre*), and n=4 mice (*Mboat7*$^{fl/fl}$*; Sox1-Cre*) from two independent litters). (C) Immunostaining for cleaved caspase-3 in E13.5 cortices of *Mboat7*$^{+/fl}$*; Sox1-Cre* and *Mboat7*$^{fl/fl}$*; Sox1-Cre*. (D) Quantitative analysis of cells positive for cleaved caspase-3 per area within 100 µm-wide-bins (n=6 embryos (*Mboat7*$^{+/fl}$*; Sox1-Cre*) and n=5 embryos (*Mboat7*$^{fl/fl}$*; Sox1-Cre*) from two independent litters). (E) Immunostaining for p-H3 in E12.5-E14.5 cortices of *Mboat7*$^{+/fl}$*; Sox1-Cre* and *Mboat7*$^{fl/fl}$*; Sox1-Cre* mice. (F) Quantitative analysis of RGCs positive for p-H3 (apical and dispersed) per area within 200 µm-wide-bins (E12.5; n=3 embryos (*Mboat7*$^{+/fl}$*; Sox1-Cre*) and n=4 embryos (*Mboat7*$^{fl/fl}$*; Sox1-Cre*), E13.5; n=6 embryos (*Mboat7*$^{+/fl}$*; Sox1-Cre*) and n=4 embryos (*Mboat7*$^{fl/fl}$*; Sox1-Cre*), E14.5; n=4 embryos (*Mboat7*$^{+/fl}$*; Sox1-Cre*) and n=4 embryos (*Mboat7*$^{fl/fl}$*; Sox1-Cre*) from two independent litters). Dotted lines show the apical surface of the cortex. (G,H) pCAGGS-mClover3 and pCAGGS-E-cadherin vectors were introduced into RGCs of *Mboat7*$^{+/fl}$*; Sox1-Cre* and *Mboat7*$^{fl/fl}$*; Sox1-Cre* mice by *in utero* electroporation at E12.5. The E13.5 cortices were immunostained for E-cadherin (G, red), p-H3 (H, red), and Sox2 (H, cyan). (I) Percentage of mClover3^{+} cells attached to apical surface in the ventricular zone within 200-µm-bins (n=5 embryos (*Mboat7*$^{+/fl}$*; Sox1-Cre*) and n=6 embryos (*Mboat7*$^{fl/fl}$*; Sox1-Cre*) from two independent litters). (J) Immunostaining for GM130 in E12.5 cortices of *Mboat7*$^{+/fl}$*; Sox1-Cre* and *Mboat7*$^{fl/fl}$*; Sox1-Cre* mice. (K) Measurement of the length of the average GM130^{+} Golgi apparatus in the ventricular zone within 50-µm-bins (n=3 embryos (*Mboat7*$^{+/fl}$*; Sox1-Cre*) and n=4 embryos (*Mboat7*$^{fl/fl}$*; Sox1-Cre*) from two independent litters). Data are shown as mean ± SEM; *p < 0.05, **p < 0.01, ***p < 0.001; unpaired two-tailed Student's *t*-test (D,I), unpaired two-tailed Welch's *t*-test (K), and multiple *t*-tests (B,F). The color of the asterisks corresponds to the color of the respective groups in the graph. Scale bars, 1 cm in (A); 100 µm in (C,E); 50µm in (G,H); 10 µm in (J).

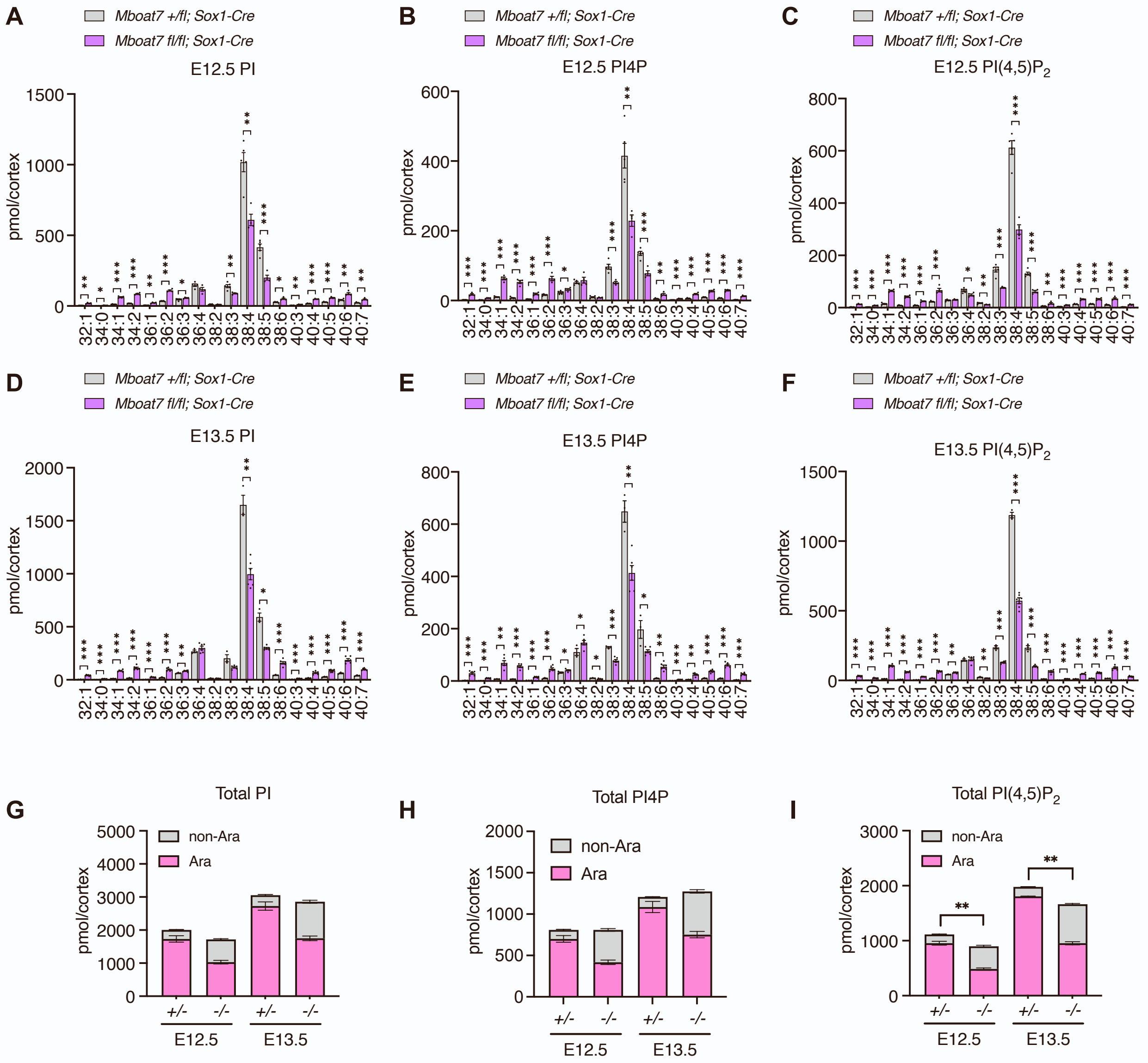

Figure S7. Molecular species and total amounts of PI, PI4P, and PI(4,5)P_2 in the cortices of neural-specific *Mboat7* KO mice at E12.5 and E13.5, related to Figure 7.

(A-C) SFC-MS/MS analysis of PI (A), PI4P (B), and PI(4,5)P_2 (C) molecular species in E12.5 cortices of *Mboat7*$^{+/fl}$*; Sox1-Cre* and *Mboat7*$^{fl/fl}$*; Sox1-Cre* mice (n=5 embryos (*Mboat7*$^{+/fl}$*; Sox1-Cre*) and n=4 embryos (*Mboat7*$^{fl/fl}$*; Sox1-Cre*) from two independent litters). (D-F) SFC-MS/MS analysis of PI (D), PI4P (E), and PI(4,5)P_2 (F) molecular species in E13.5 cortices of *Mboat7*$^{+/fl}$*; Sox1-Cre* and *Mboat7*$^{fl/fl}$*; Sox1-Cre* mice (n=3 embryos (*Mboat7*$^{+/fl}$*; Sox1-Cre*) and n=6 embryos (*Mboat7*$^{fl/fl}$*; Sox1-Cre*) from two independent litters). Peak areas were normalized by the area of the internal standard (37:4 PI, PI4P, or 37:4 PI(4,5)P_2). (G-I) Quantification of total amounts of PI (G), PI4P (H), and PI(4,5)P_2 (I) in E12.5 and E13.5 cortices of *Mboat7*$^{+/fl}$*; Sox1-Cre* (+/-) and and *Mboat7*$^{fl/fl}$*; Sox1-Cre* (-/-) mice using SFC-MS/MS based method (E12.5: n=5 embryos (*Mboat7*$^{+/fl}$*; Sox1-Cre*) and n=4 embryos (*Mboat7*$^{fl/fl}$*; Sox1-Cre*); E13.5: n=3 embryos (*Mboat7*$^{+/fl}$*; Sox1-Cre*) and n=6 embryos (*Mboat7*$^{fl/fl}$*; Sox1-Cre*) from two independent litters. Ara and non-Ara indicate arachidonic acid-containing and non-arachidonic acid-containing species, respectively. Data are shown as mean ± SEM; *$p < 0.05$, **$p < 0.01$, ***$p < 0.001$; multiple t-tests.

Time	Genotype	*Mboat7*$^{+/fl}$	*Mboat7*$^{fl/fl}$	*Mboat7*$^{+/fl}$; *Sox1-Cre*	*Mboat7*$^{fl/fl}$; *Sox1-Cre*
1 week	Number of animals<br>%	5<br>25.0	5<br>25.0	7<br>35.0	3<br>15.0
2 week	Number of animals<br>%	8<br>42.1	4<br>21.1	6<br>31.6	1<br>5.2
3 week	Number of animals<br>%	24<br>38.1	19<br>30.2	20<br>31.7	0<br>0.0*

Table S1. Neural-specific *Mboat7* KO mice die within a month after birth, related to Figure 7.
Genotypes of litters from intercrosses of *Mboat7*$^{+/fl}$*; Sox1-Cre* with *Mboat7*$^{fl/fl}$ mice. DNA was extracted from the ear of each mouse and subjected to PCR analysis to determine the genotype.
The p values were calculated using the chi-square test. *p =0.000069.

Species	Q1	Q3
25:0 PC	636.5	184.0
30:0 PC	706.3	184.0
32:0 PC	734.5	184.0
32:1 PC	732.5	184.0
34:0 PC	762.5	184.0
34:1 PC	760.5	184.0
34:2 PC	758.5	184.0
36:1 PC	788.5	184.0
36:2 PC	786.5	184.0
36:3 PC	784.5	184.0
36:4 PC	782.5	184.0
38:4 PC	810.5	184.0
38:5 PC	808.5	184.0
38:6 PC	806.5	184.0

Species	Q1	Q3
25:0 PE	594.5	453.5
34:0 PE	720.5	579.5
34:1 PE	718.5	577.5
34:2 PE	716.5	575.5
36:1 PE	746.5	605.5
36:2 PE	744.5	603.5
36:3 PE	742.5	601.5
36:4 PE	740.5	599.5
38:3 PE	770.5	629.5
38:4 PE	768.5	627.5
38:5 PE	766.5	625.5
38:6 PE	764.5	623.5
40:4 PE	796.5	655.5
40:5 PE	794.5	653.5
40:6 PE	792.5	651.5
40:7 PE	790.5	649.5

Species	Q1	Q3
25:0 PS	636.3	549.3
34:0 PS	762.5	675.5
34:1 PS	760.5	673.5
36:0 PS	790.5	703.5
36:1 PS	788.5	701.5
36:2 PS	786.5	699.5
38:3 PS	812.5	725.5
38:4 PS	810.5	723.5
40:3 PS	840.5	753.5
40:4 PS	838.5	751.5
40:5 PS	836.5	749.5
40:6 PS	834.5	747.5
42:5 PS	864.5	777.5

Species	Q1	Q3
25:0 PI	711.5	241.0
32:1 PI	807.5	241.0
34:0 PI	837.5	241.0
34:1 PI	835.5	241.0
34:2 PI	833.5	241.0
36:1 PI	863.5	241.0
36:2 PI	861.5	241.0
36:3 PI	859.5	241.0
36:4 PI	857.5	241.0
38:2 PI	889.5	241.0
38:3 PI	887.5	241.0
38:4 PI	885.5	241.0
38:5 PI	883.5	241.0
38:6 PI	881.5	241.0
40:3 PI	915.5	241.0
40:4 PI	913.5	241.0
40:5 PI	911.5	241.0
40:6 PI	909.5	241.0
40:7 PI	907.5	241.0

Phospholipids	DP	EP	CE	CXP
PC	171	10	37	14
PE	116	10	31	12
PS	-100	-10	-36	-15
PI	-100	-10	-56	-9

Table S2. MRM transitions of PC,PE, PS, and PI (LC-MS/MS), related to Figure 7.
PC and PE species are detected in positive ion mode. Injection volume: 2 µl, Dwell time: 5.4 msec, Curtain Gas (CUR): 30 psi, Temperature (TEM): 700 ºC , Source Gas 1 (GS1): 30 psi, Source Gas 2 (GS2): 50 psi, Ion Source (IS): 5500 V, Collision Gas (CAD) : 7 (arbitrary units)
PS and PI species are detected in negative ion mode. Injection volume: 10µl injection, Dwell time: 5.4 msec, Curtain Gas (CUR): 30 psi, Temperature (TEM): 700 ºC , Source Gas 1 (GS1): 30 psi, Source Gas 2 (GS2): 70 psi, Ion Source (IS): -4500 V, Collision Gas (CAD) : 7 (arbitrary units)

Species	Q1	Q3
37:4 PI4P	995.6	613.6
32:1 PI4P	931.6	549.6
34:0 PI4P	961.6	579.6
34:1 PI4P	959.6	577.6
34:2 PI4P	957.6	575.6
36:1 PI4P	987.6	605.6
36:2 PI4P	985.6	603.6
36:3 PI4P	983.6	601.6
36:4 PI4P	981.6	599.6
38:2 PI4P	1013.6	631.6
38:3 PI4P	1011.6	629.6
38:4 PI4P	1009.6	627.6
38:5 PI4P	1007.6	625.6
38:6 PI4P	1005.6	623.6
40:3 PI4P	1039.6	657.6
40:4 PI4P	1037.6	655.6
40:5 PI4P	1035.6	653.6
40:6 PI4P	1033.6	651.6
40:7 PI4P	1031.6	649.6

Species	Q1	Q3
37:4 PI(4,5)P_2	1103.6	613.6
32:1 PI(4,5)P_2	1039.6	549.6
34:0 PI(4,5)P_2	1069.6	579.6
34:1 PI(4,5)P_2	1067.6	577.6
34:2 PI(4,5)P_2	1065.6	575.6
36:1 PI(4,5)P_2	1095.6	605.6
36:2 PI(4,5)P_2	1093.6	603.6
36:3 PI(4,5)P_2	1091.6	601.6
36:4 PI(4,5)P_2	1089.6	599.6
38:2 PI(4,5)P_2	1121.6	631.6
38:3 PI(4,5)P_2	1119.6	629.6
38:4 PI(4,5)P_2	1117.6	627.6
38:5 PI(4,5)P_2	1115.6	625.6
38:6 PI(4,5)P_2	1113.6	623.6
40:3 PI(4,5)P_2	1147.6	657.6
40:4 PI(4,5)P_2	1145.6	655.6
40:5 PI(4,5)P_2	1143.6	653.6
40:6 PI(4,5)P_2	1141.6	651.6
40:7 PI(4,5)P_2	1139.6	649.6

Species	Q1	Q3
37:4 PI	887.6	613.6
32:1 PI	823.6	549.6
34:0 PI	853.6	579.6
34:1 PI	851.6	577.6
34:2 PI	849.6	575.6
36:1 PI	879.6	605.6
36:2 PI	877.6	603.6
36:3 PI	875.6	601.6
36:4 PI	873.6	599.6
38:2 PI	905.6	631.6
38:3 PI	903.6	629.6
38:4 PI	901.6	627.6
38:5 PI	899.6	625.6
38:6 PI	897.6	623.6
40:3 PI	931.6	657.6
40:4 PI	929.6	655.6
40:5 PI	927.6	653.6
40:6 PI	925.6	651.6
40:7 PI	923.6	649.6

Phospholipids	DP	EP	CE	CXP
PI4P	130	10	33	13
PI(4,5)P_2	150	10	37	17
PI	100	10	33	15

Table S3. MRM transitions of PI4P, PI(4,5)P2, and PI (SFS-MS/MS), related to Figure 7.
PI4P, PI(4,)P_2, and PI species are detected in positive ion mode. Injection volume: 10 µl, Dwell time: 21msec, CUR: 10 psi, TEM: 500 ºC , GS1: 30 psi, GS2: 70 psi, IS: 4500 V, CAD: 7 (arbitrary units)

Data S1

Data S1. Original, uncropped immunoblots, related to Figure 1.

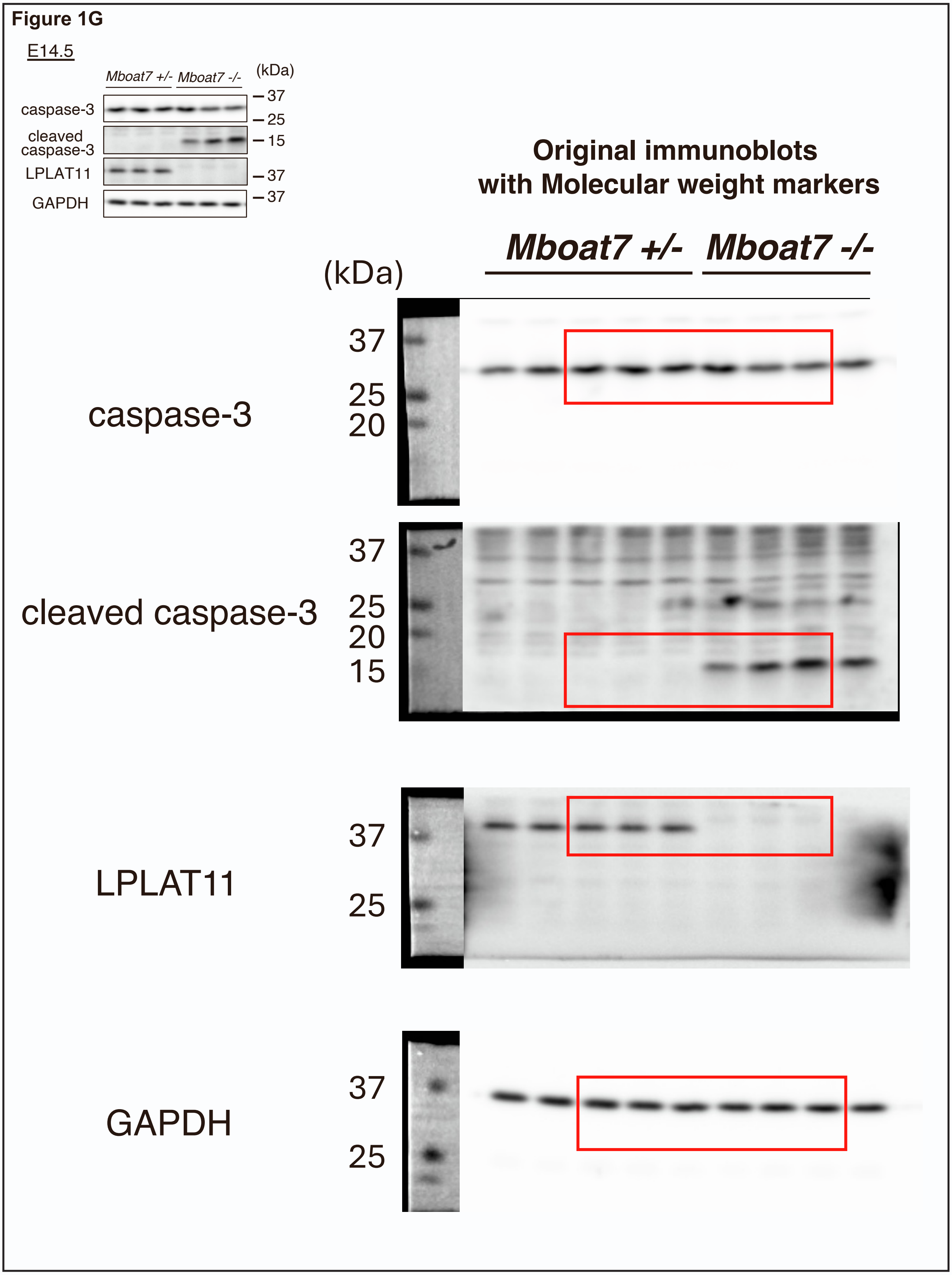

Supplement: Document S1. — Figures S1–S7, Tables S1–S3, and Data S1 [file mmc1.pdf]
